# Direct electrosynthesis and separation of ammonia and chlorine from waste streams via a stacked membrane-free electrolyzer

**DOI:** 10.1038/s41467-024-52830-4

**Published:** 2024-09-30

**Authors:** Jianan Gao, Qingquan Ma, Zhiwei Wang, Bruce E. Rittmann, Wen Zhang

**Affiliations:** 1 Department of Civil and Environmental Engineering, New Jersey Institute of Technology, Newark, NJ US; 2 State Key Laboratory of Pollution Control and Resource Reuse, Shanghai Institute of Pollution Control and Ecological Security, Tongji Advanced Membrane Technology Center, School of Environmental Science and Engineering, Tongji University, Shanghai, China; 3 Biodesign Swette Center for Environmental Biotechnology, Arizona State University, Tempe, AZ US; 4 Department of Chemical & Materials Engineering, New Jersey Institute of Technology, Newark, NJ US

**Keywords:** Pollution remediation, Electrocatalysis, Sustainability, Electrocatalysis

## Abstract

Electrosynthesis, a viable path to decarbonize the chemical industry, has been harnessed to generate valuable chemicals under ambient conditions. Here, we present a membrane-free flow electrolyzer for paired electrocatalytic upcycling of nitrate (NO_3_^−^) and chloride (Cl^−^) to ammonia (NH_3_) and chlorine (Cl_2_) gases by utilizing waste streams as substitutes for traditional electrolytes. The electrolyzer concurrently couples electrosynthesis and gaseous-product separation, which minimizes the undesired redox reaction between NH_3_ and Cl_2_ and thus prevents products loss. Using a three-stacked-modules electrolyzer system, we efficiently processed a reverse osmosis retentate waste stream. This yielded high concentrations of (NH_4_)_2_SO_4_ (83.8 mM) and NaClO (243.4 mM) at an electrical cost of 7.1 kWh per kilogram of solid products, while residual NH_3_/NH_4_^+^ (0.3 mM), NO_2_^−^ (0.2 mM), and Cl_2_/HClO/ClO^−^ (0.1 mM) pollutants in the waste stream could meet the wastewater discharge regulations for nitrogen- and chlorine-species. This study underscores the value of pairing appropriate half-reactions, utilizing waste streams to replace traditional electrolytes, and merging product synthesis with separation to refine electrosynthesis platforms.

## Abstract (web summary)

Electrosynthesis offers a greener alternative to traditional chemical production by utilizing renewable energy and waste streams. Herein, the authors report a membrane-free electrolyzer that efficiently produces and separates ammonia and chlorine from wastewater.

## Introduction

Conventional chemical industries depend on fossil fuels and emit large amounts of greenhouse gases^1^. In contrast, electrosynthesis is an emerging redox platform that can achieve more environmentally compatible chemical production that is more amenable to using renewable energy sources (i.e., solar and wind)^2,3^. For example, renewable feedstock -- air, water, CO_2_, and derivatives of biomass -- have been converted to portable fuels, such as NH_3_ and C_2_H_5_OH, and to important industrial chemicals, such as Cl_2_, CO, H_2_, C_2_H_4_, and CH_3_OH^2^.

Today, electrolytes such as tetrahydrofuran, toluene, and inorganic salts are employed to enhance electron transfer in electrosynthesis processes^3–8^. These inputs increase the costs of input materials and for treating secondary wastes. Conversely, wastewaters that contain dissolved contaminants could be utilized as electrolytes, offering a globally abundant and underexploited resource to tap into^9–11^. Approximately 2.2 × 10^15 ^L of wastewater, constituting 54% of total freshwater withdrawals, is generated annually across municipal, agricultural, and industrial sectors^12^. For instance, the electrocatalytic valorization of chlorinated organic water pollutants to ethene was recently proven feasible^13^. Minimizing the costs of input materials, avoiding secondary contaminants, and electrochemically valorizing waste elements will offset wastewater treatment costs^14,15^.

An important example is the conversion of NO_3_^−^ and Cl^−^ ions to NH_3_ and Cl_2_ gases, which are chemicals produced globally at approximately 182 million and 88 million metric tons per year, respectively^16–20^. NO_3_^−^ and Cl^−^ are commonly present in industrial wastewater, such as ion-exchange brines, which may contain 150 mM NO_3_^−^ and 5 wt% NaCl^21,22^. Electrocatalytic conversion of nitrate to ammonia, which has been demonstrated^14,23^, involves cathodic nitrate reduction (Eq. 1) coupled to an anodic reaction such as water oxidation (Eq. 2)^9,24^. Similarly, industrial chlorine gas (Cl_2_) is primarily produced by the chlor-alkali process, which consists of an anodic chlorine-evolution reaction paired with a hydrogen (H_2_)-evolution reaction (Eqs. 3 and 4)^25,26^.

1 $${{{\rm{Cathode}}}}-1:\quad{{{{{\rm{NO}}}}}_{3}}^{-}+{{{{\rm{6H}}}}}_{2}{{{\rm{O}}}}+{{{{\rm{8e}}}}}^{-}\to {{{{\rm{NH}}}}}_{3}+{{{{\rm{9OH}}}}}^{-}$$

2 $${{{\rm{Anode}}}}-1:\quad{{{{\rm{2H}}}}}_{2}{{{\rm{O}}}}\to {{{{\rm{O}}}}}_{2}+{{{{\rm{4H}}}}}^{+}+{{{{\rm{4e}}}}}^{-}$$

3 $${{{\rm{Anode}}}}-2:\quad{{{{\rm{2Cl}}}}}^{-}\to {{{{\rm{Cl}}}}}_{2}+{{{{\rm{2e}}}}}^{-}$$

4 $${{{\rm{Cathode}}}}-2:\quad{{{{\rm{2H}}}}}_{2}{{{\rm{O}}}}+{{{{\rm{2e}}}}}^{-}\to {{{{\rm{H}}}}}_{2}+{{{{\rm{2OH}}}}}^{-}$$

While today’s processes separately generate O_2_ and H_2_, it makes sense to couple the nitrate-to-ammonia conversion with chlorine evolution. To synchronize NH_3_/Cl_2_ production, the rapid reaction between NH_3_ and Cl_2_ (a rate constant of *c.a*. 4.2 × 10^6 ^M^−1^·s^−1^) must be prevented^27^. One approach involves the use of ion-selective or -exchange membranes to separate the cathode and anode and their respective NH_3_ and Cl_2_ productions^28–30^. However, the substantial initial cost of membrane material costs—~ 24% of the electrolyzer-stack costs—and problems related to the durability and requisite maintenance of the membranes lead to high capital and operating costs of the electrolyzer^31–33^. Therefore, it would be of value to devise a process free from ion selective/exchange membrane for the synchronous production and extraction of NH_3_/Cl_2_ products, as long as product purity, product yield rate, and efficiency are optimally balanced.

Recently, membrane modules integrated with hydrophobic gas-diffusion layers have emerged as effective tools for gaseous compound extraction (e.g., CH_4_ and H_2_)^34^, delivery of CO_2_ and N_2_^35^, and hybrid processes. When these hydrophobic interfaces operate below their liquid entry pressure, they establish a triphasic boundary, working as a liquid water barrier, but allowing the passage of gases. In addition, by integrating another electrocatalyst layer into the membrane module and referencing Fick’s law, we note that Faradaic reactions involving proton consumption or production result in localized pH extremes: alkaline conditions (>11.5) at the cathode and acidic conditions (<2.5) at the anode, even with modest current densities of 5 mA·cm^−2^ (Supplementary Fig. 1). Such pH environments can promote the formation of gaseous NH_3_ and Cl_2_ at their respective primary interfaces^16,36^. By directing separation at the membrane-water junction instead of at the traditional gas-liquid interface, we hypothesize that bespoke chemical reactions on the membrane surface might achieve the production and separation of NH_3_/Cl_2_ products while simultaneously minimizing product losses.

**Fig. 1:**
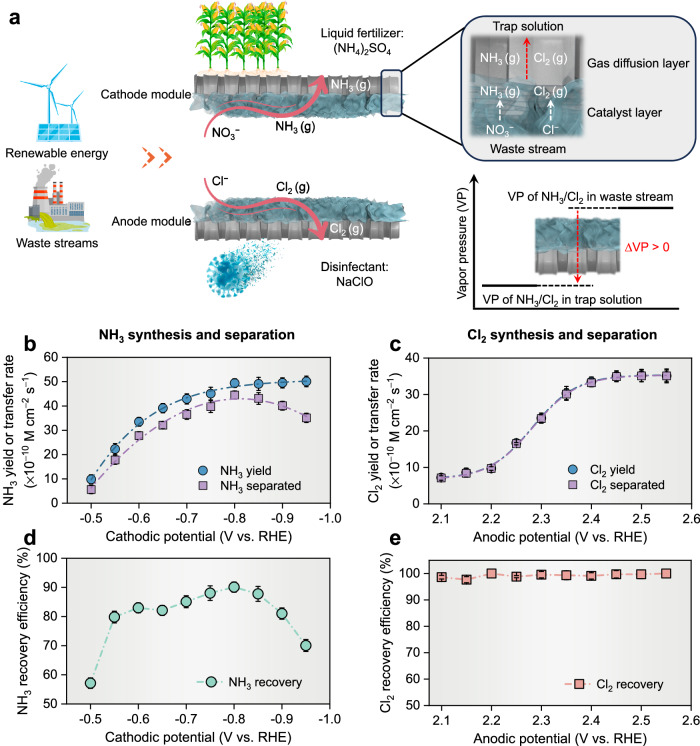
Concept and verification of synergistic electrosynthesis and separation of NH_3_ and Cl_2_. **a** Schematic of the electrochemical NH_3_ and Cl_2_ production under ambient conditions using renewable energy and waste stream. **b**, **c** The yield and separation rates of NH_3_ and Cl_2_ as a function of applied potentials on cathodic and anodic electrode assemblies, respectively. **d**, **e** The recovery efficiencies of yield NH_3_ and Cl_2_ as a function of applied potentials on cathodic and anodic electrode assemblies, respectively. No iR compensation was applied. The synthetic waste stream is 25 mM NO_3_^−^ or 25 mM Cl^−^ mixed with 0.1 M Na_2_SO_4_ (pH = 7.0 ± 0.1) to simulate co-existing ions in the waste stream. The error bars represent the standard deviations from triplicate tests.

In this report, we present an electrified membrane-free electrolyzer featuring gas-extraction electrodes for synchronous NH_3_/Cl_2_ production and extraction (Fig. 1a). First, we demonstrated that the electrode assembly combining electrocatalyst layer and gas exchange layer can effectively balance the production and separation of NH_3_ and Cl_2_. Building on this, we integrated the gas-extraction electrodes into a flow-type, membrane-free electrolyzer, achieving synchronous electrosynthesis and separation of NH_3_ and Cl_2_ with high product purity, high yield rates, and minimal product loss. Our comprehensive investigation delves into the electrochemical conversion pathways, the homogeneous redox dynamics of nitrogen- and chlorine-derived species, and the mechanisms facilitating the selective extraction of NH_3_ and Cl_2_, thereby enriching our understanding of the complex interactions within the system. We then successfully implemented a stacked electrolyzer comprised of three modules and a geometric electrode area of up to 300 cm². This system efficiently processed the actual reverse osmosis retentate waste stream, resulting in high product concentrations ((NH_4_)_2_SO_4_: 83.8 mM, NaClO: 243.4 mM) and low residual intermediates/products (NH_3_/NH_4_^+^: 0.3 mM, NO_2_^−^: 0.2 mM, Cl_2_/HClO/ClO^−^: 0.1 mM). This research not only opens avenues for the upscaling of electrosynthesis platforms using waste streams in place of traditional electrolytes but also provides critical insights into product synthesis and separation pathways.

## Results

### Electrode assembly design and basic performance evaluation

To achieve synergistic electrosynthesis and separation of NH_3_ and Cl_2_ from waste streams, the key is to develop electrode assemblies with high catalytic activity and gas-transfer rate. Metallic copper and ruthenium oxide were selected as model electrocatalysts for nitrate reduction reaction (NO_3_RR) and chlorine evolution reaction (CER) due to their rapid reduction/oxidation kinetics of NO_3_^−^ and Cl^−^ towards NH_3_ and Cl_2_^22,37^. The electrocatalysts (hierarchical Cu or RuO_2_ particles) were further immobilized to a carbon-polytetrafluoroethylene (PTFE)-based gas diffusion layer to obtain a gas-extraction electrode (Supplementary Fig. 2). Scanning electron microscopy (SEM) demonstrated the uniform loading of the electrocatalytic layer (Supplementary Fig. 3), while the X-ray diffraction (XRD) patterns confirmed the successful fabrication of Cu or RuO_2_ dominated electrocatalytic layer (Supplementary Fig. 4).

We then examined separately the electrosynthesis and separation rates of the gas extraction electrodes for NH_3_ and Cl_2_. A synthetic medium-strength waste stream containing either 25 mM NO_3_^−^ or 25 mM Cl^−^ (pH = 7.0 ± 0.1) was employed. We posited that spontaneous stripping of NH_3_ and Cl_2_ would occur at the gas diffusion layer, propelled by a concentration gradient in local vapor pressure across the electrode module (Fig. 1a). As depicted in Fig. 1b, increasing the cathodic potential from − 0.50 to − 0.80 V vs. RHE corresponded to a surge in the NH_3_ yield rate, from 9.8 ± 1.1 to 49.4 ± 0.7 × 10^−10^ M-NH_3_·cm^−2^ · s^−1^, accompanied by an upswing in the NH_3_ transfer rate. Beyond − 0.80 V vs. RHE, the NH_3_ yields stabilized between 49.1 ± 2.7 to 50.1 ± 2.2 × 10^−10^ M-NH_3_·cm^−2^ · s^−1^, but its transfer rate kept diminishing. This phenomenon can be ascribed to the enhanced Faradaic Efficiency (FE) for H_2_ production at elevated cathodic potentials (Supplementary Fig. 5). The concurrent efflux of H_2_ competes for the gas transfer channels with NH_3_, resulting in a diminished NH_3_ transfer rate. Meanwhile, for Cl_2_, Fig. 1c illustrates an S-shaped relationship with applied anodic potential, where Cl_2_ yield and transfer rates fluctuated between 34.9 ± 1.3 to 35.2 ± 1.7 × 10^−10^ M-Cl_2_·cm^−2^ · s^−1^) was reached when the anode potential was greater than 2.45 V vs. RHE, beyond which the rate was constrained by the limited mass transfer of Cl^−^. Our findings underscore that NH_3_ and Cl_2_ yields were influenced by the applied potential, which also controlled the product transfer rates. Furthermore, the NH_3-_separation efficiency (defined as the ratio of the molar amount of separated NH_3_ to the total molar amount of NH_3_ produced) had a volcano-shaped response to the cathodic potential, peaking at −0.80 V vs RHE with 90 ± 2% efficiency (Fig. 1d). In contrast, the Cl_2_ separation efficiency performed stable with an average value of 99 ± 1% across a broad potential range (Fig. 1e). Thus, when the NO_3_RR and CER reactions were synchronized, anodic potential could be used to sensitively match the cathode potential. The high product-separation efficiency hints at the feasibility of a unified NH_3_ and Cl_2_ electrosynthesis-separation in one membrane-free electrolyzer.

### The synchronous electrosynthesis and separation of NH_3_ and Cl_2_

**Fig. 2:**
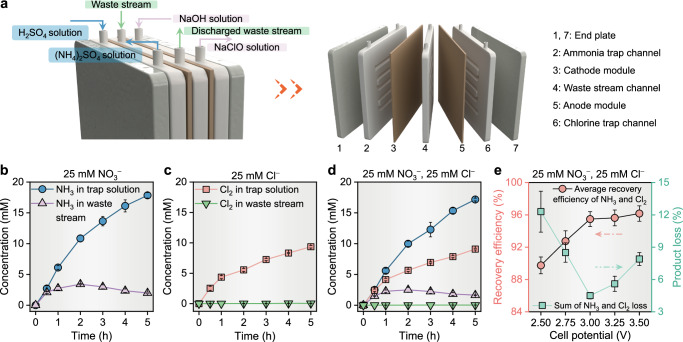
The performance of synchronous electrosynthesis and separation of NH_3_ and Cl_2_ of the flow-type membrane-free electrolyzer. **a** Schematics and configuration of this flow-type membrane-free electrolyzer for electrochemical synthesis and in situ recovery of ammonium sulfate and hypochlorous acid from waste streams. **b**, **c** Single-electrosynthesis for NH_3_ (**b**) and Cl_2_ (**c**): the concentrations of NH_3_ or Cl_2_ in the trap solution and the waste stream as a function of reaction time by feeding 25 mM NO_3_^−^ or 25 mM Cl^−^ mixed with 0.1 M Na_2_SO_4_ (pH = 7.0 ± 0.1). **d** Co-electrosynthesis for NH_3_ and Cl_2_: the concentrations of NH_3_ and Cl_2_ in the trap solution and the waste stream as a function of reaction time by feeding 25 mM NO_3_^−^ and 25 mM Cl^−^ mixed with 0.1 M Na_2_SO_4_ (pH = 7.0 ± 0.1). **e** The average recovery efficiencies and the sum of the product loss of NH_3_ and Cl_2_ at different total cell potentials. The error bars represent the standard deviations from triplicate tests.

We then investigated the performance of simultaneous NH_3_ and Cl_2_ electrosynthesis and separation. The gas-extraction electrodes were incorporated into a flow-type membrane-free electrolyzer, which consisted of an ammonia trap channel (circulating pH 1.0 ± 0.1 H_2_SO_4_ solution), a chlorine trap channel (circulating pH 13.0 ± 0.1 NaOH solution), and a waste stream channel (Fig. 2a). The physical installation diagram of the electrolyzer is shown in Supplementary Fig. 6. The gas extraction electrodes separated two trap channels from the middle waste stream channel. Nitrate and chlorite underwent interfacial electrochemical reactions at the electrodes and were converted into gaseous products (NH_3_ and Cl_2_) with synchronous transfer across the gas diffusion layers into the trap electrolytes. We hypothesized that rapid extraction rates for NH_3_ and Cl_2_ could obviate their contact within the sewage stream, which would effectively thwart the undesirable reactions between NH_3_/NH_4_^+^ and reactive chlorine species such as HClO/ClO^−^ in the electrolyte, leading to N_2_ and Cl^−^ as final products. The stripped NH_3_ and Cl_2_ were chemically converted to (NH_4_)_2_SO_4_ and NaClO, respectively, within their designated trap channels.

Based on potential-controlled experiments, various constant cell potentials were utilized, in contrast to employing individually controlled cathodic or anodic potentials, to balance the NH_3_ and Cl_2_ production and separations. Initially, we determined the baseline concentrations of NH_3_ and Cl_2_ at this cell potential without the interactions between products. As demonstrated in Fig. 2b, c, after 5 h of single-electrosynthesis with the synthetic waste stream of either 25 mM NO_3_^−^ or 25 mM Cl^−^, the concentrations of NH_3_ and Cl_2_ in their respective trap solutions reached 17.9 ± 0.5 mM and 9.4 ± 0.2 mM. Subsequently, we introduced a synthetic mixed waste stream containing 25 mM NO_3_^−^ and 25 mM Cl^−^ to monitor the product concentrations during co-electrosynthesis of NH_3_ and Cl_2_. The typical I-t curve and pH variations were illustrated in Supplementary Fig. 7, confirming the stability of the system. As shown in Fig. 2d, the final NH_3_ and Cl_2_ concentrations in the trap solutions were 17.1 ± 0.2 mM and 9.3 ± 0.4 mM, respectively, which were close to their baseline values. Notably, the final NH_3_ concentration in the waste stream during co-electrosynthesis (1.6 ± 0.1 mM) was lower than that during single-electrosynthesis (2.0 ± 0.2 mM), while Cl_2_ was even undetectable in the waste stream for both processes. This reduced NH_3_ concentration in the waste stream could be attributed to the oxidation of NH_3_/NH_4_^+^ by Cl_2_/HClO/ClO^−^ species. When the cell potential increased from 2.5 V to 3.5 V, the average recovery efficiency (pink data points) for NH_3_ and Cl_2_ improved from 90 ± 1% to 96 ± 1%, as Fig. 2e and Supplementary Fig. 8 indicate. The total loss of produced NH_3_ and Cl_2_ (green data points) varied between 12 ± 2% to 5 ± 1%, with the minimum value located at 3.0 V. These results validate the membrane-free electrolyzer’s effectiveness in co-electrosynthesizing NH_3_ and Cl_2_ with high efficiency and acceptable product loss. Furthermore, the interaction between residual nitrogen and chloride species in the waste stream resulted in the formation of N_2_ and Cl^−^ as final products, further minimizing the residual products such as NH_3_/NH_4_^+^ and Cl_2_/HClO/ClO^−^.

### Probing the mechanism of NH_3_/Cl_2_ separation on reducing product loss

**Fig. 3:**
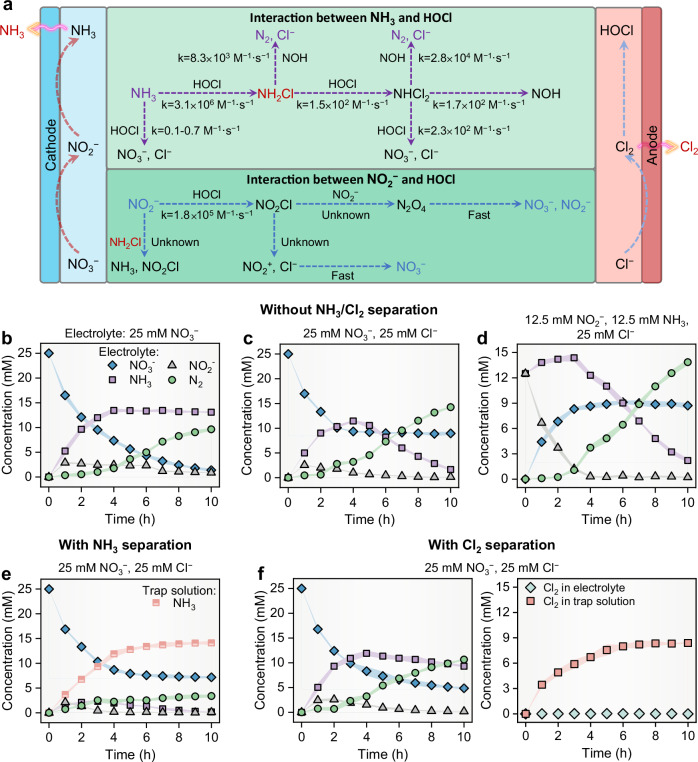
Mechanism analysis. **a** The major heterogeneous and homogeneous redox reactions within the electrolyzer. **b**–**f** the nitrogen species and active chlorine species evolution over reaction time when the electrolytes in the reaction chamber contained 0.1 M Na_2_SO_4_ mixed with (**b**) 25 mM NO_3_^−^; (**c**) 25 mM NO_3_^−^, 25 mM Cl^−^; (**d**) 12.5 mM NO_2_^−^, 12.5 mM NH_3_, 25 mM Cl^−^, pH = 12.0 ± 0.1; (**e**) 25 mM NO_3_^−^, 25 mM Cl^−^; (**f**) 25 mM NO_3_^−^, 25 mM Cl^−^. All experiments were carried out under a total cell potential of 3.0 V. The shadow area in the figure represents the error scale. The error scales represent the standard deviations from triplicate tests.

To gain insights into the effect of NH_3_/Cl_2_ separation on reducing product loss, we conducted a series of control experiments by varying the concentrations/ratios of nitrogen and chloride species in the feed electrolyte, with and without the incorporation of separation operations. The major heterogeneous and homogeneous redox reactions within the electrolyzer are shown in Fig. 3a and summarized in Supplementary Table 1^38^. As shown in Fig. 3b, c, the introduction of Cl^−^ ions resulted in an observable increase in the remaining NO_3_^−^ concentration (blue data points), from 1.4 ± 0.3 mM to 9.0 ± 0.3 mM, and a corresponding decrease in the final NH_3_ (purple data points) concentration, from 13.1 ± 0.2 mM to 1.7 ± 0.2 mM. The concentration of N_2_ (green data points), encompassing both dissolved and vaporized forms, increased from 9.6 ± 0.3 mM to 14.3 ± 0.1 mM. This calculation was based on the disparity between the input nitrate nitrogen and the nitrogen species retained in the solution. After 4 h, the stability of NH_3_ concentration, despite a decreasing NO_3_^−^ concentration, is attributed to the direct oxidation of NH_3_ under alkaline conditions^38^, as indicated by the electrolyte pH rise above 11.5. The amplified N_2_ concentration when introducing Cl^−^ is attributed to the more rapid reaction kinetics toward N_2_ (4.2 × 10^6 ^M^−1^·s^−1^) compared to NO_3_^−^ (0.1 − 0.7 M^−1^·s^−1^) in the context of NH_3_/NH_4_^+^ interaction with HClO/ClO^–^^27,38^. As the rate-limiting species for NO_3_^−^ reduction, the average NO_2_^−^ concentration within 10 h electrolysis (gray data points) reduced from 1.9 ± 0.1 mM to 0.9 ± 0.1 mM, when Cl⁻ was present.

Subsequent experiments aimed to trace the NO_2_^−^ conversion pathways (e.g., conversion to NO_3_^−^ or NH_3_) and to ascertain the oxidation priorities of active chlorine species with NO_2_^−^ and NH_3_. To this end, equal amounts of NO_2_^−^ and NH_3_ were introduced together to the electrolyte, and the evolution of subsequent nitrogen species was monitored. Figure 3d indicates that during the initial 3 h, the NO_2_^−^ concentration decreased from 12.5 mM to 1.2 ± 0.2 mM and was mainly oxidized to NO_3_^−^ that increased from 0 mM to 8.3 ± 0.2 mM. Meanwhile, the N_2_ and NH_3_ concentrations increased from 0 mM and 12.5 mM to 1.1 ± 0.2 mM and 14.4 ± 0.5 mM, respectively. After 3-h, when NO_2_^−^ was nearly depleted, NH_3_ oxidation became dominant, as indicated by the reduced NH_3_ concentration from 14.4 ± 0.4 mM to 2.2 ± 0.2 mM and the increased N_2_ concentration from 1.1 ± 0.2 mM to 13.4 ± 0.4 mM. From a reaction kinetic standpoint, NH_3_ has multi-step conversions with rate constants spanning from 1.7 × 10^2^ to 3.1 × 10^6^ M^−1^ · s^−1^ and is considerably more vulnerable to HOCl-induced oxidation than NO_2_^−^ that has multi-step conversions with measured rate constant for only one step by far (1.8 × 10^5^ M^−1^ · s^−1^)^39–42^. The preferential reaction of NO_2_^−^ with HOCl can be explained by the breakpoint chlorination mechanism in NH_3_, which occurs when the HOCl to NH_3_ mole ratio gradually reaches 1.5^38^. The continuous consumption of HOCl by NO_2_^−^ prevents the system from reaching this breakpoint chlorination ratio. During HOCl-mediated NH_3_ oxidation to N_2_ and NO_3_^−^, chloramine intermediates react with NO_2_^−^, converting back to NH_3_ as end products^42^. This process further influences the dynamics of NH_3_/HOCl interactions. The main interference arises from HOCl that converts NO_2_^−^ to NO_3_^−^ at a kinetic rate five orders of magnitude faster than chloramine reactions due to the low HOCl concentration condition within the electrolyzer^43^.

We further assessed the influence of the separation of NH_3_ and Cl_2_ from the electrolyte on the final product formation. Attaching an ammonia trap channel next to the gas-permeable cathode (Fig. 3e) separated over 99% of the produced NH_3_ from the electrolyte channel (red data points). Consequently, the NO_3_^−^-conversion efficiency increased by 11%, and nitrogen loss evidenced by N_2_ formation also decreased by 76%. A similar experiment was conducted by incorporating a single chlorine trap channel (without the use of the ammonia trap channel), which yielded a modest improvement of the NO_3_^−^ conversion efficiency by 46% and the reduced N_2_ loss by 25% according to the results in Fig. 3f. Although ~ 100% of the generated Cl_2_ was extracted from electrolyte, after 5 h electrolysis, the Cl_2_ concentration in the chlorine trap solution (7.5 ± 0.5 mM) was still lower than that in Fig. 2c (9.1 ± 0.1 mM), where the separation of NH_3_ and Cl_2_ occurred simultaneously. This observation implies that the average Cl_2_ recovery efficiency of 99% across a broad anodic potential range in Fig. 1e may be misleading. The Cl_2_ extraction kinetics rate is not fast enough to efficiently separate all produced Cl_2_ at the anodic interface, which consequently causes the residual HClO and ClO^−^ in the waste stream. These residual species are likely to engage in side reactions with NH_3_ and NO_2_^−^, resulting in the formation of Cl^−^. Therefore, the optimal electrosynthesis and separation performance of the membrane-free electrolyzer required synchronous extraction processes for NH_3_ and Cl_2_. Isolating ammonia or chlorine gas alone was inadequate to mitigate product loss.

### Large-scale electrosynthesis using a stacked electrolyzer system

The practical implementation and performance (e.g., productivity and product concentration, energy consumption, and intermediates/products residual) of this membrane-free electrolyzer are highly affected by complex water chemistry of the feeding waste stream (e.g., concentrations and ratios of NO_3_^−^ and Cl^−^ ions), which should be considered. Supplementary Figs. 9 and 10 show the time-resolved evolution of nitrogen and chlorine species for synthetic waste streams with different nitrate and chloride concentrations or ratios. The pH of all synthetic waste streams was controlled to be 7.0 ± 0.1. The relevant current density and NH_3_/Cl_2_ FE are summarized in Supplementary Table 2.

**Fig. 4:**
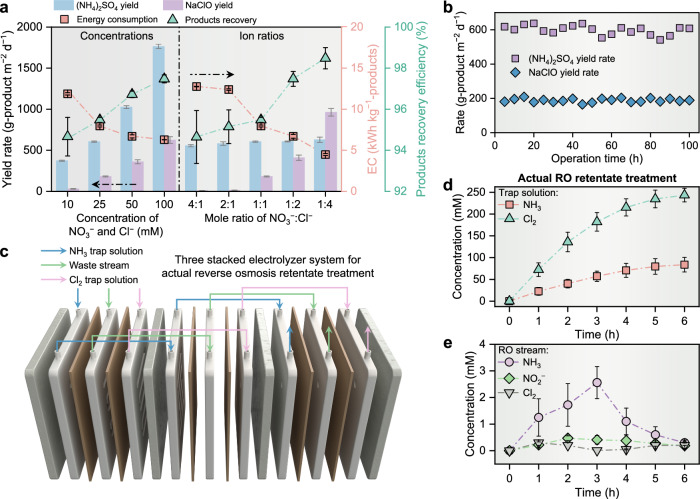
Scalable electrosynthesis by using stacked flow-type membrane-free electrolyzer system. **a** Comparation of products ((NH_4_)_2_SO_4_ and NaClO) yield rate, energy consumption for production, and products recovery efficiency. Testing conditions are shown in the experimental section (i.e., pH = 7.0 ± 0.1, Na_2_SO_4_ = 0.1 M; NO_3_^−^ = 10–100 mM, Cl^−^ = 6.25–100 mM, cell potential: 3.0 V). **b** Cycle performance of the membrane-free electrolyzer without changing the cathodic/anodic modules at 3.0 V total cell potential, pH = 7.0 ± 0.1, 0.1 M Na_2_SO_4_, 25 mM NO_3_^−^, 25 mM Cl^−^. Each cycle undergoes 5 h with >85% NO_3_^−^ is converted to NH_3_, and > 90% NH_3_ is separated from the electrolyte. **c** Illustration of stacked electrolyzer system consisting of three modules. **d**, **e** The performance of stacked electrolyzer system for actual reverse osmosis (RO) retentate treatment. Specifically, the concentration of recovery NH_3_ and Cl_2_ in trap solutions and residual concentrations of NH_3_/NH_4_^+^, Cl_2_/HClO/ClO^−^, and NO_2_^−^ in the RO stream as a function of operation time under a total cell potential of 3.0 V were recorded. The error bars represent the standard deviations from triplicate tests.

The yield rate and energy consumption for the production of (NH_4_)_2_SO_4_ and NaClO, along with NH_3_/Cl_2_ recovery efficiencies from the waste stream, were measured and summarized for the different feed-solution chemistries. We first increased the same molar concentrations of NO_3_^−^ and Cl^−^ in the feed solution from 10 mM (typical for most industrial wastewater^44^) to 100 mM found in brine wastewater from ion exchange or reverse osmosis processes^21^, under a fixed cell potential of 3.0 V. The left side of Fig. 4a shows the high concentration of NO_3_^−^ and Cl^−^ led to a fast reaction kinetics of NO_3_^−^-to-NH_3_ and Cl^−^-to-Cl_2_ conversions and thus decreased the overall energy consumption from 11.9 ± 0.1 to 6.3 ± 0.1 kWh·kg^−1^-products, accompanied by the increased NH_3_/Cl_2_ average recovery efficiency (95 ± 1% to 97 ± 0%) and two product generation rates or fluxes (373 ± 7 to 1763 ± 25 g-(NH_4_)_2_SO_4_·m^−2^·d^−1^ and 30 ± 3 to 625 ± 14 g-NaClO·m^−2^·d^−1^, respectively). This result indicates that the NH_3_/Cl_2_ transfer across the gas-diffusion layer was not a limiting factor and should be higher than the gaseous product generation rate on the catalyst layer under the NO_3_^−^/Cl^−^ concentration in common waste streams.

We then changed the mole ratio of NO_3_^−^ and Cl^−^ ions concentration from 4:1 to 1:4 under the same NO_3_^−^ concentration (25 mM). The data in the right side of Fig. 4a show that the (NH_4_)_2_SO_4_ generation rates and NH_3_/Cl_2_ recovery efficiencies remained relative stable between 556 ± 13 to 628 ± 19 g-(NH_4_)_2_SO_4_·m^−2^·d^−1^ and 95 ± 1% to 98 ± 1% as the ratio of NO_3_^−^/Cl^−^ decreased. Using the same high feed Cl^−^ concentration (100 mM), a NaClO generation rate of 962 ± 46 g-NaClO·m^−2^·d^−1^ was obtained for the mole ratio of NO_3_^−^/Cl^−^ of 1:4 and outperformed that (625 ± 40 g-NaClO·m^−2^·d^−1^) when NO_3_^−^/Cl^−^ = 1:1. That could be attributed to the high feed NO_3_^−^ concentration leading to a high NH_3_ yield, which in turn caused more un-separated NH_3_ to react with the active chlorine in the waste stream^38^, causing more Cl_2_ loss. In conclusion, this membrane-free electrolyzer could consistently produce and recover NH_3_/Cl_2_ across various waste stream conditions.

Extended electrolysis experiments reveal that the electrocatalyst and gas diffusion layer were stable for over 100 h of synchronous production of (NH_4_)_2_SO_4_ and NaClO with an average yield rate of 598 g-(NH_4_)_2_SO_4_·m^−2^·d^−1^ and 182 g-NaClO·m^−2^·d^−1^, respectively (Fig. 4b). To enhance productivity, we expanded the area of individual electrode module from 9 cm^2^ to 50 cm^2^ and scaled up the reactor from a single module to a configuration of three tandem stacked modules (Fig. 4c), which constitutes a cumulative geometric electrode area of 300 cm^2^. The cell potential for each module was consistently maintained at 3.0 V. The real reverse osmosis retentate from the Yuma Desalination Plant in Arizona was further used as the feed waste stream, containing average concentrations of NO_3_^−^ and Cl^−^ at ~ 11.8 mM and 54.1 mM (pH 6.6), respectively, alongside other co-existing contaminants such as Ca^2+^, Mg^2+^, Na^+^, SO_4_^2−^ et al. A comprehensive analysis of the feed waste stream composition is provided in Supplementary Table 3. To boost the production of (NH_4_)_2_SO_4_ and NaClO, 0.2-liter solutions of H_2_SO_4_ and NaOH were utilized as the trapping solutions for NH_3_ and Cl_2_, respectively. As shown in Fig. 4d, following a 6-h operation period, the stacked electrolyzer system yielded 83.8 ± 16.7 mM of (NH_4_)_2_SO_4_ and 243.4 ± 15.6 mM of NaClO, accompanied by the nitrogen and chlorine average utilization efficiencies of 71% and 45%, respectively. The electrical consumption for the simultaneous production of (NH_4_)_2_SO_4_ and NaClO was calculated at 7.1 kWh per aggregate kilogram of solid products. Concurrently, the residual concentrations of NH_3_/NH_4_^+^, Cl_2_/HClO/ClO^−^, and NO_2_^−^ in the treated reverse osmosis retentate were found at 0.32 ± 0.19 mM, 0.06 ± 0.02 mM, and 0.15 ± 0.08 mM (Fig. 4e), respectively. These values are below the relevant nitrogen- and chlorine-species regulatory limits for wastewater discharges into receiving water bodies. For instance, the World Health Organization (WHO) and the US Environmental Protection Agency (EPA) have not established a Maximum Contaminant Level (MCL) for ammonia, but common environmental limits for ammonia in surface water typically range between 0.02–2.32 mM. In addition, the MCL for free chlorine in drinking water, as stipulated by the US EPA, is set at 0.11 mM. The MCL for nitrite in drinking water is defined as 0.21 mM N by WHO^45^ and 0.07 mM by the US EPA^46^. These findings underscore the viability of the stacked, membrane-free electrolyzer system for industrial-scale applications.

### Economic analysis and operation viability

A simple techno-economic analysis (TEA) was conducted to evaluate the profitability of this approach to synthesize ammonium sulfate and sodium hypochlorite using renewable energy sources (e.g., wind power, solar power, bioenergy, and hydroelectric) and the synthetic feed wastewater^47^. The TEA calculation was based on electricity cost of 5¢·kWh^−1^ and the current market prices of ammonium sulfate ($533·ton^−1^) and sodium hypochlorite ($958·ton^−1^ for 60% purity)^48,49^. The computational contour plot depicted in Supplementary Fig. 11 clearly illustrates the impact of improvements in energy-related parameters (kg-(NH_4_)_2_SO_4_/NaClO·kWh^−1^), along with a reduction in the unit cost of electricity or a combination of both, in significantly mitigating the production costs of (NH_4_)_2_SO_4_ and NaClO. The outcomes demonstrate the sensitivity of energy-related parameters to the NO_3_^−^/Cl^−^ concentrations and ratios. Based on the laboratory-scale data and after deducting the associated electricity costs, the average profit attainable per metric ton for the obtained (NH_4_)_2_SO_4_ and NaClO from synthetic waste streams with variable water chemistry parameters is projected to be $1550, as depicted in Supplementary Fig. 12. For the real RO stream, the projected profit is estimated at $2364 when scaled up to an industrial scale electrolyzer. This financial metric underscores the potential economic viability of this electrosynthesis process from the waste stream.

To produce different ammonium salts, we employed HNO_3_, H_3_PO_4_, and a mixed acid solution comprising HNO_3_, H_2_SO_4_, and H_3_PO_4_ in a 1:1:1 molar ratio for NH_3_ capture. The results in Supplementary Fig. 13 reveal comparable NH_3_ capture efficiencies across different acid solutions, underscoring the system’s adaptability and flexibility in producing various ammonium salts. To avoid the use of hazardous chemicals, in situ acid/alkaline production via water electrolysis reaction was achieved with a proton exchange membrane-separated electrolyzer, featuring a stainless-steel mesh cathode and DSA anode, which generated the acid and alkaline solutions from a 0.1 M Na_2_SO_4_ feed. As shown in Supplementary Fig. 14, two types of operations were tested: two-stage (where acid/alkaline solutions are generated first and then used for NH_3_/Cl_2_ capture) and single-stage (where acid-base solutions are generated simultaneously while capturing NH_3_/Cl_2_). Although NH_3_ capture efficiency remained consistent, significant Cl_2_ product loss (85%) was observed in the single-stage operation (Supplementary Fig. 15). This loss is likely because NH_4_^+^ cannot be readily oxidized at the anode, whereas OCl^−^ can be reduced to Cl^−^ at the cathode^16,38^.

We further conducted experiments in a single-pass mode without recirculating the waste stream storage tank, detailed in Supplementary Figs. 16, 17. At flow rates of 5 mL·min^−1^ and 25 mL·min^−1^, the yields of (NH_4_)_2_SO_4_ reached 64.51 ± 3.21 mM and 23.43 ± 1.19 mM, respectively, after 5 h, with NH_3_ separation efficiencies of 54% and 71%. The discharged waste stream contained NO_2_^−^ and NH_3_ concentrations ranging from 0.21 ± 0.02 mM to 1.07 ± 0.06 mM and 0.96 ± 0.06 mM to 5.48 ± 0.18 mM, respectively. The Cl_2_ separation efficiencies exceeded 99%, maintaining residual active chlorine concentrations in the discharged waste stream below 0.01 mM throughout. To ensure compliance with regulatory limits, careful control of the flow rate is essential. These findings provide critical data for decision-makers and stakeholders to assess the economic benefits and potential applications of this membrane-free electrolyzer in chemical synthesis with waste streams.

## Discussion

This study showcases the simultaneous separation and recovery of NH_3_ and Cl_2_ from waste streams containing NO_3_^−^ and Cl^−^ using a flow-type membrane-free electrolyzer. Within the electrolyzer, three primary stages are involved: (1) electrochemical conversion of NO_3_^−^ and Cl^−^ ions into NH_3_ and Cl_2_; (2) vaporization of NH_3_ and Cl_2_ at the respective basic and acidic interfaces of the cathode and anode; and (3) interfacial extraction of NH_3_ and Cl_2_ at the electrode surface. The pairing of nitrate-reduction-to-ammonia with chloride-oxidation-to-chlorine evolutions eliminated undesired by-products, such as H_2_ and O_2_. The specially designed gas extraction electrode concurrently coupled electrosynthesis and product extraction, achieving the simultaneous generation and separation of NH_3_ and Cl_2_ on the same interface, thereby preventing significant product loss caused by redox reactions between NH_3_ and Cl_2_. Scale-up electrosynthesis using a stacked electrolyzer system with a geometric electrode area of up to 300 cm² and real reverse osmosis retentate waste stream was proven to be feasible. This work highlights the promise of combining NH_3_ and Cl_2_ production/separation using a straightforward electrolyzer configuration.

Future research should include the electrosynthesis and separation of a more diverse array of bulk and fine chemicals. In addition, future studies should explore integrating pre-concentration processes for low-concentration waste streams and optimizing reactor or catalyst layer designs, such as zero-gap electrolyzers, flow-through electrodes, or coupled porous adsorption materials to overcome mass transfer limitations. Electrosynthesis based on waste streams presents a cost-effective alternative to traditional waste removal processes, maximizing the value extracted from complex, abundant wastewater resources. Its on-site deployment at wastewater treatment facilities or pollution sources supports the circular economy, promotes energy sustainability, and enables zero liquid discharge. This approach offers substantial economic, environmental, and societal advantages.

## Methods

### Materials and reagents

Copper sulfate pentahydrate (≥ 99%), ruthenium dioxide nanoparticles (99.95%), sulfuric acid (98%), isopropanol (99.6%), sodium nitrate (98.8%), sodium chloride (99%), sodium sulfate (99%), sodium hydroxide (97%), nitric acid (69%–70%), hydrochloric acid (36.5%–38%), sulfamic acid (99%), p-aminobenzene sulphanilamide (98%), N-(1-Naphthyl) ethylenediamine dihydrochloride (96%), phosphoric acid (85%), salicylic acid (≥ 99%), sodium citrate dihydrate (≥ 99%), sodium hypochlorite (5.65%-6%), and sodium nitroferricyanide (99%) were obtained from Thermo Fisher Scientific and used without further purification. The DPD-free chlorine reagent powder pillow was obtained from HACH Company. AvCarb GDS2230 substrate, Teflon PTFE DISP 30 Fluoropolymer Dispersion, Nafion 117 membrane, Vulcan XC 72 carbon black, and Nafion D-521 dispersion were purchased from Fuel Cell Store. Deionized (DI) water (18.2 MΩ cm) was applied throughout all experiments in this research.

### Fabrication of the Cu dendrite gas extraction electrode

A copper (Cu) dendrite electrocatalyst layer was deposited on a commercial carbon-based substrate (AvCarb GDS2230). The substrate consists of a carbon fiber layer (PTFE treated) and a carbon-based micro-porous layer (Supplementary Fig. 2). To enhance the anti-wetting properties of the gas extraction electrode, the substrate was further coated with another non-conductive PTFE hydrophobic layer on the PTFE treated carbon fiber layer side via air-brush spray using a 10 wt% PTFE solution and calcination operation (obtained by diluting Teflon PTFE DISP 30 Fluoropolymer Dispersion by DI water). The catalyst was deposited on the carbon-based micro-porous layer via an electrodeposition process in a typical three-electrode system. Briefly, a CHI 150 saturated calomel electrode (SCE), an IrO_2_ − RuO_2_/Ti electrode (obtained from Yunxuan Metallic Materials Co. Ltd., China) (total size: 7 cm × 7 cm, area exposed to the electrolyte: 3 cm × 3 cm), and the substrate (total size: 4 cm × 4 cm, area exposed to the electrolyte: 3 cm × 3 cm) were used as reference electrode, counter electrode, and working electrode, respectively. The working electrode and counter electrode chambers were filled with 20 mL 0.1-M CuSO_4_·5H_2_O solution (prepared by pH = 2.0 ± 0.1 DI water, adjusted by 1 M H_2_SO_4_) and 20 mL 0.1-M Na_2_SO_4_ solution (pH = 7.0 ± 0.1), which were separated with a proton-exchange membrane (Nafion 117, total size: 7 cm × 7 cm, 183 μm in thickness, immersed in 0.1 M Na_2_SO_4_ solution overnight before use). All electrolytes were stored at room temperature, approximately 20 °C, and were utilized or disposed of within one week. Before catalyst deposition, the microporous layer side of each substrate was infiltrated with 200 μL isopropanol to improve the substrate’s surface wettability. A constant potential (– 0.743 V vs SCE for 700 s) was applied to the working electrode by a CH Instruments 700E Potentiostat. After electrodeposition, the obtained Cu dendrite catalyst layer was rinsed with DI water and then dried in a 50 °C vacuum for 5 h. The catalyst loading was controlled to be 2.30 ± 0.05 mg·cm^−2^, calculated by dividing the mass difference of the substrate before and after catalyst application by the electrode area exposed to the electrolyte.

### Fabrication of the RuO_2_ anodic gas extraction electrode

The RuO_2_ electrocatalyst layer was deposited on the same pretreated substrate via air-brush painting of the catalyst ink. The catalyst ink was prepared by mixing 10 mg of RuO_2_ nanoparticles, 5 mg of Vulcan XC 72 carbon black, and 100 μL Nafion solution (D521 Nafion Dispersion at 5 wt%, containing ~ 4 mg Nafion) in 3.9 mL isopropanol. After sonication (50-60 Hz and 230 W) for 1 h, 2 mL of the catalyst ink was uniformly sprayed using an airbrush onto the substrate (total size: 4 cm × 4 cm)^50^. The RuO_2_ catalyst layer-coated electrode was further air-dried overnight before testing. The catalyst loading was controlled to be 0.30 ± 0.03 mg·cm^−2^, calculated by dividing the mass difference of the substrate before and after catalyst application by the total electrode size.

### Electrocatalyst-coated electrode characterization

The morphology and chemical composition of a prepared aqueous gas-extraction electrode was analyzed by JSM-7900F field emission scanning electron microscope (FE-SEM) (JEOL, Japan). The crystalline structures of the electrocatalysts were investigated by X-ray powder diffractometer (XRD) performed on a Philips, EMPYREAN, PANalytical Almelo with a Co Ka radiation (λ = 1.789 Å).

### Electrolyzer setup and operation

Supplementary Fig. 6 shows the major assembly procedure of the NH_3_ trap channel, waste stream channel, and Cl_2_ trap channel (all with length and width of 30 mm × 30 mm and depth of 10 mm in the flow cell, except for the waste stream channel with a depth of 20 mm) with the corresponding gas-extraction electrodes. Silicone gaskets (30 mm × 30 mm exposure window) ensured adequate sealing between each channel or end plate. All channels featured identical-sized inlets and outlets (4 mm OD; 2 mm ID) for electrolyte flow. The Cu-dendrite cathode and the RuO_2_ anode separated the middle waste stream channel from the NH_3_ trap channel and the Cl_2_ trap channel, respectively. The electrocatalyst-coated side of the aqueous gas extraction electrodes faced the waste stream, whereas the PTFE gas diffusion layer side faced the NH_3_ or Cl_2_ trap channels. NH_3_ and Cl_2_ gases were extracted from the wastewater through the gas extraction electrodes’ gas diffusion layer into the NH_3_ or Cl_2_ trap channels, respectively, due to the vapor pressure gradient of NH_3_ and Cl_2_ gases.

To evaluate synchronous electrosynthesis and separation of NH_3_ and Cl_2_ from waste stream, 50 ml synthetic wastewater solutions with different NO_3_^−^ and Cl^−^ concentrations (pH = 7.0 ± 0.1, Na_2_SO_4_ = 0.1 M NO_3_^−^ = 10–100 mM, Cl^−^ = 6.25–100 mM) were prepared and circulated between the waste stream channel and a feed tank at a flow rate of 25 mL·min^−1^ using a peristaltic pump (MASTERFLEX L/S, Avantor, Radnor, US). Synthetic wastewater was prepared by adding target amounts of NaNO_3_, NaCl, and Na_2_SO_4_ into 500 mL DI water to achieve the desired concentrations. The trap solutions were recirculated between the storage tank and the NH_3_ trap channel with 50 mL pH = 1.0 ± 0.1 solution or the Cl_2_ trap channel with 50 mL pH = 13.0 ± 0.1 solution at a flow rate of 25 mL·min^−1^. Trap solutions of varying pH levels were prepared by adding 1 M H_2_SO_4_ or 1 M NaOH into 500 mL DI water and monitoring the pH value to achieve the desired acidity or alkalinity. All synthetic wastewater solutions and trap solutions were stored at room temperature, ~20 °C, and were utilized or disposed of within one week.

The individual NH_3_ or Cl_2_ electrosynthesis and separation were performed under a constant cell potential using a CH Instruments 700E Potentiostat at room temperature (~25 °C) and atmospheric pressure. These experiments were operated in a three-electrode configuration with Cu dendrite gas extraction electrode, RuO_2_ anodic gas extraction electrode, and SCE serving as working electrode, counter electrode, and reference electrode, respectively. The potential measured was calibrated into a reversible hydrogen electrode (RHE) by:

5 $${E}_{RHE}={E}_{SCE}+0.241{{{\rm{V}}}}+0.0591\times {{{\rm{pH}}}}$$

For synchronous electrosynthesis and separation of NH_3_ and Cl_2_ experiments, a DC power supply was used with total cell potentials ranging from 2.5 V to 3.5 V. The Cu dendrite gas extraction electrode and RuO_2_ anodic gas extraction electrode served as cathode and anode, respectively. To record the current values of the electrolyzer operated under typical recycle mode and single pass mode, a CH Instruments 700E Potentiostat was utilized. These experiments were operated in a two-electrode system with the Cu dendrite gas extraction electrode and RuO_2_ anodic gas extraction electrode serving as the working and counter electrodes, respectively. A constant cell potential of 3.0 V was maintained throughout these experiments. The collected current values are shown in Supplementary Figs. 7, 17.

The major products within the electrolyzer may have included nitrate-N, nitrite-N, ammonia-N, and free chlorine (hypochlorous acid and hypochlorite ion), which were analyzed using ultraviolet-visible (UV-vis) spectrophotometry as detailed in Supplementary Information, Section 10)^9^. To ascertain the ammonia source, control experiments were performed by adding or removing NO_3_^−^ to/from the synthetic wastewater solution. The findings confirmed that the produced ammonia originated exclusively from nitrate in the catholyte rather than from ammonia-containing pollutants present in synthetic reagent raw materials, air, or human breath, used for the electrocatalytic layer. The relevant yield rate, separation efficiency, and energy consumption of obtained products were also calculated as shown in Supplementary Information, Section 11.

The real reverse osmosis retentate from the Yuma Desalination Plant in Arizona was also used as the feed waste stream to carry out the electrosynthesis experiment. The detailed composition analysis of the actual RO retentate was listed in Supplementary Table 3.

## Supplementary information

Supplementary Information

Peer Review File

## Source data

Source Data

## Data Availability

The data underlying the findings of this study are provided in the main text and Supplementary Information. Additional data related to the results discussed are available from the corresponding authors upon reasonable request. Source data are also provided as a Source Data file. Source data are provided in this paper.
