# Supplementary material for: Direct electrosynthesis and separation of ammonia and chlorine from waste streams via a stacked membrane-free electrolyzer
Source: Nat Commun. 2024 Sep 30;15:8455. doi: 10.1038/s41467-024-52830-4 (PMC11443043; doi:10.1038/s41467-024-52830-4)
Supplement: Supplementary file 1 — Supplementary Information [file 41467_2024_52830_MOESM1_ESM.pdf]

Supplementary Information

for

Direct electrosynthesis and separation of ammonia and chlorine from wastewater streams via economically viable membrane-free flow electrolyzer

Jianan Gao [1], Qingquan Ma [1], Zhiwei Wang [2], Bruce E. Rittmann [3], Wen Zhang [1, 4*]

[1] *Department of Civil and Environmental Engineering, New Jersey Institute of Technology, Newark NJ, 07102, the United States*

[2] *State Key Laboratory of Pollution Control and Resource Reuse, Shanghai Institute of Pollution Control and Ecological Security, Tongji Advanced Membrane Technology Center, School of Environmental Science and Engineering, Tongji University, Shanghai 200092, China*

[3] *Biodesign Swette Center for Environmental Biotechnology, Arizona State University, Tempe, AZ 85287-5701, the United States*

[4] *Department of Chemical & Materials Engineering, New Jersey Institute of Technology, Newark NJ, 07102, the United States*

Corresponding Author

**e-mail:* wen.zhang@njit.edu

Summary: 26 pages, 17 figures, 3 table

Part S1. Electrode Interfacial pH calculation.

Cathodic/anodic interfacial pH calculation. The cathodic and anodic reactions cause an increasing ($pH^* \geq pH^b$) and decreasing ($pH^* \leq pH^b$) interfacial pH gradient toward the electrode surface, respectively. The transport fluxes of H^+ and OH^-, represented as currents, can be derived from Fick's law and are presented as follows:

$$j_{cathodic} = (1000Fm_H 10^{-pH^b})(1-10^{pH^b-pH^*}) + (1000Fm_{OH}10^{pH^b-pK_w})(10^{pH^*-pH^b}-1) \quad \text{(S1)}$$

$$j_{anodic} = (1000Fm_{OH}10^{b-pK_w})(1-10^{pH^*-pH^b}) + (1000Fm_H 10^{-pH^b})(10^{pH^b-pH^*}-1) \quad \text{(S2)}$$

where pH^b and pH^* are the bulk solution pH and interfacial pH, pK_w is the logarithm of the ionization constant of water. j is the overall response current ($A \cdot m^{-2}$). F is the Faraday constant (96485 $C \cdot mol^{-1}$), $m_H = D_H/\delta_N$ ($m/\cdot s^{-1}$), $m_{OH} = D_{OH}/\delta_N$ (m/s), D_H and D_{OH} are the diffusion coefficients of H^+ and OH^- ($m^2 \cdot s^{-1}$). The diffusion layer thickness (δ_N) of H^+ and OH^- are 24.5 and 21.5 μm as calculated by Levich equation.[1]

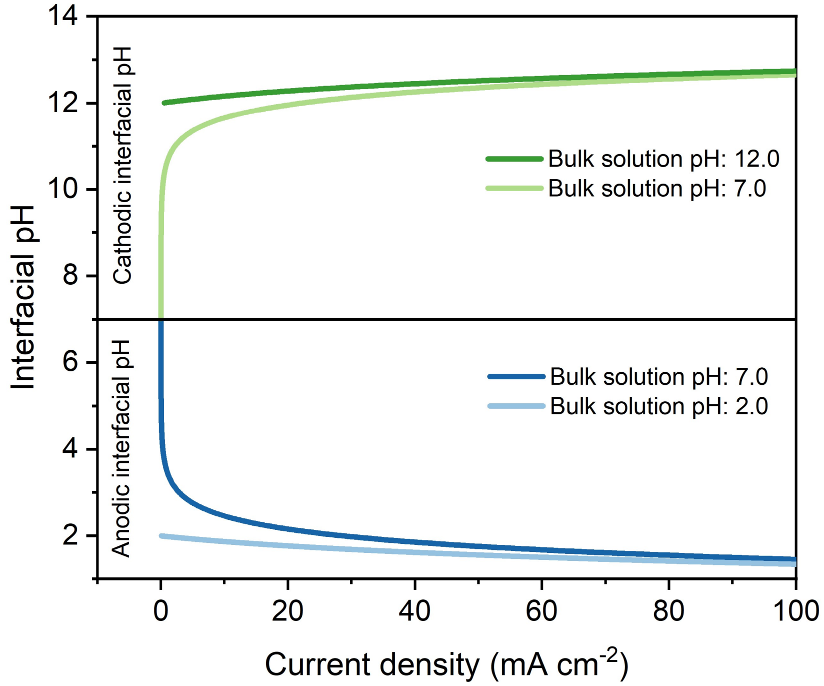

Figure S1. The relationship between cathodic/anodic interfacial pH and response current density under different bulk solution pHs. The local pH near cathode and anode was calculated as reported elsewhere.[2,3]

Part S2. Characterization of electrode assembly.

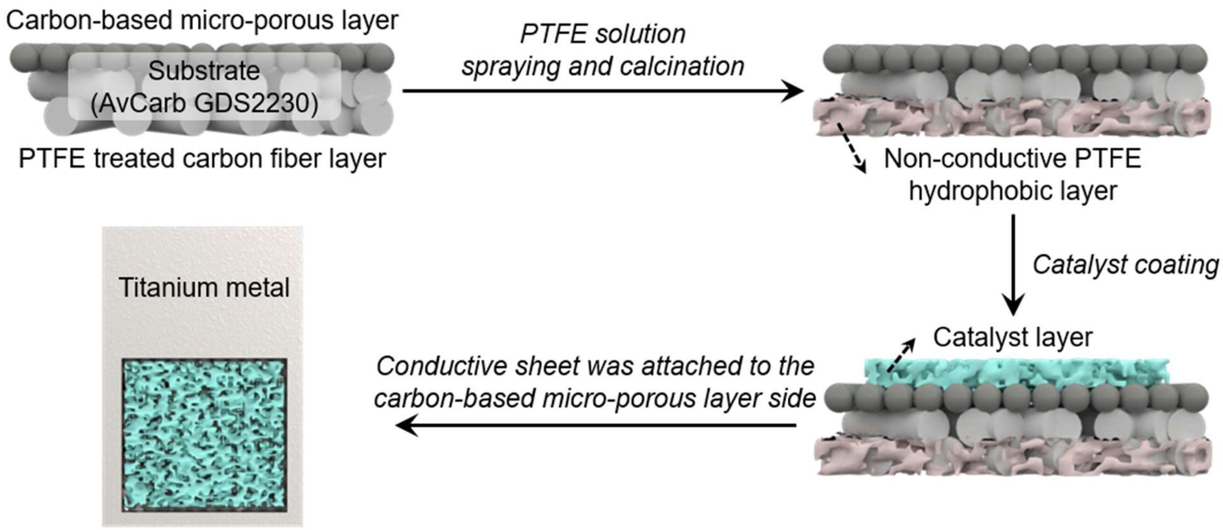

Figure S2. The illustration of the pretreatment of substrate, catalyst coating, and connection between conductive sheet and obtained electrode.

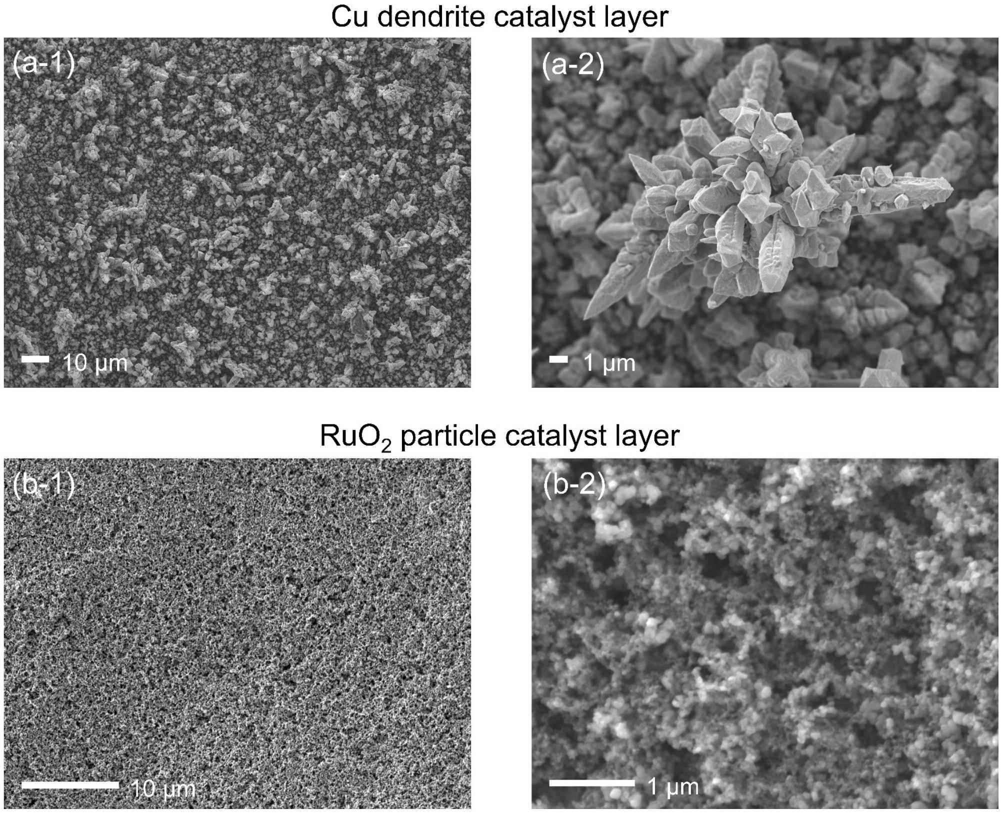

Figure S3. SEM of (a) Cu dendrite catalyst layer and (b) RuO_2 particle catalyst layer.

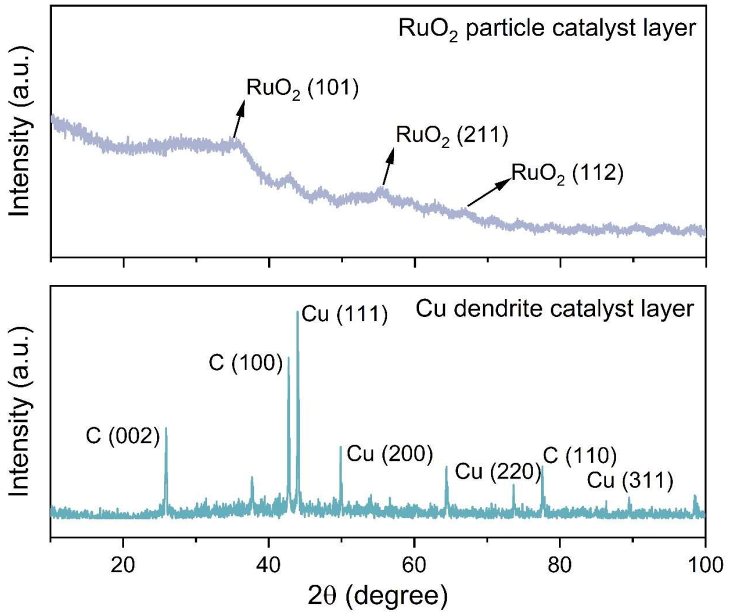

Figure S4. XRD patterns of Cu catalyst and RuO_2 catalyst.

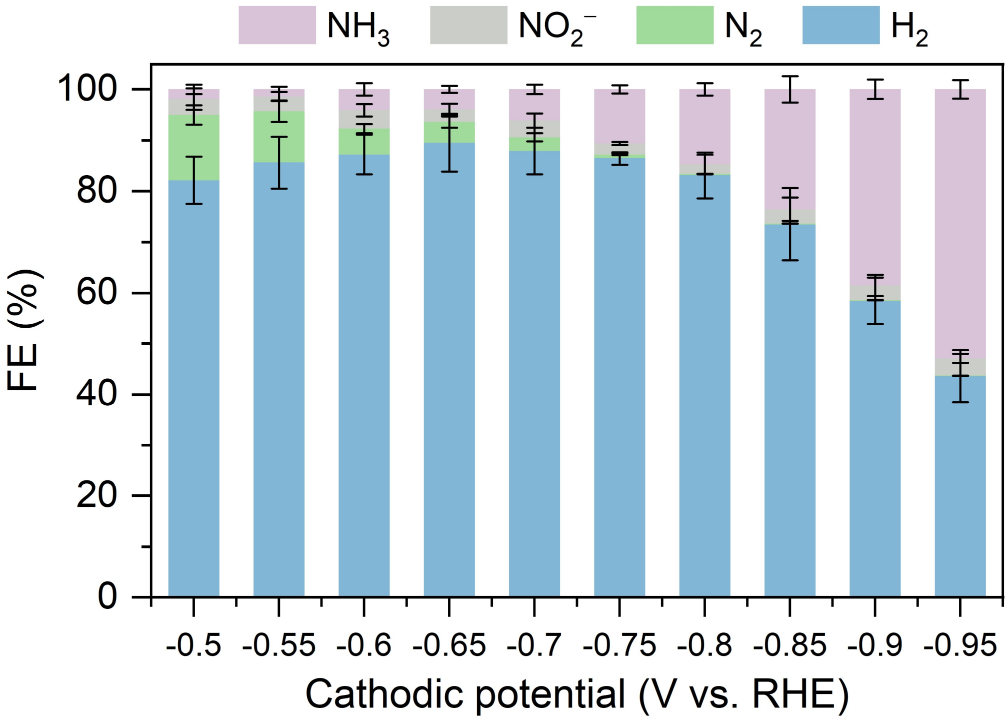

Figure S5. The Faradaic efficiency of various products under different cathodic potentials. The error bars represent the standard deviations from triplicate tests.

Part S3. Physical installation diagram of the continuous membrane-free electrolyzer.

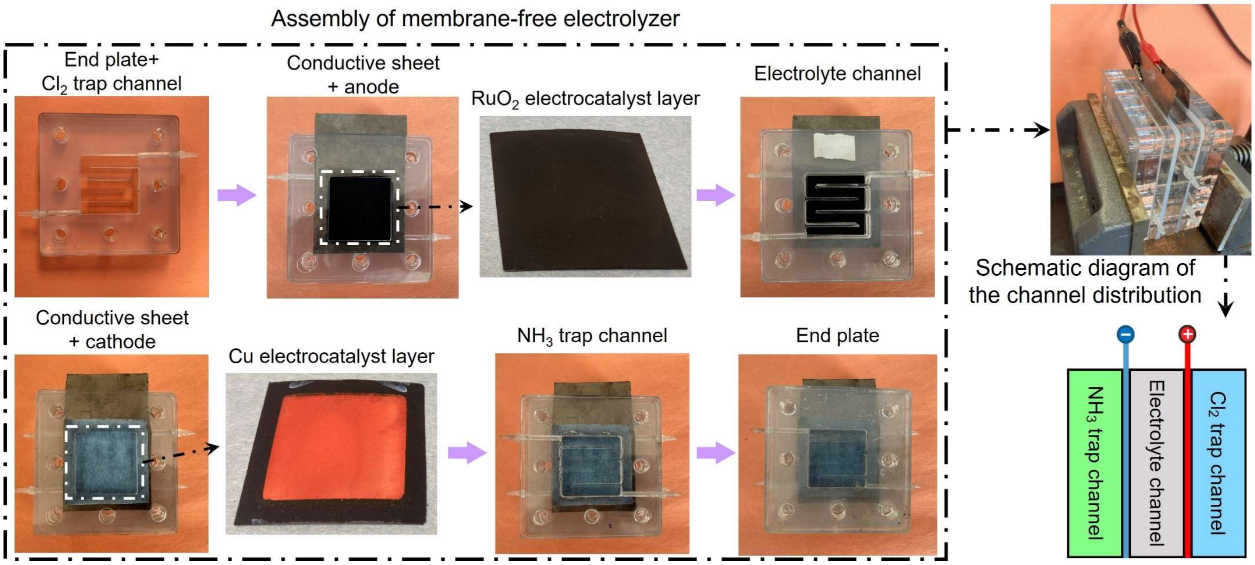

Figure S6. Illustration of the continuous membrane-free electrolyzer used for ammonium sulfate and hypochlorous acid from wastewater streams.

Part S4. Detailed N-species evolution of membrane-free electrolyzer with different total cell potentials.

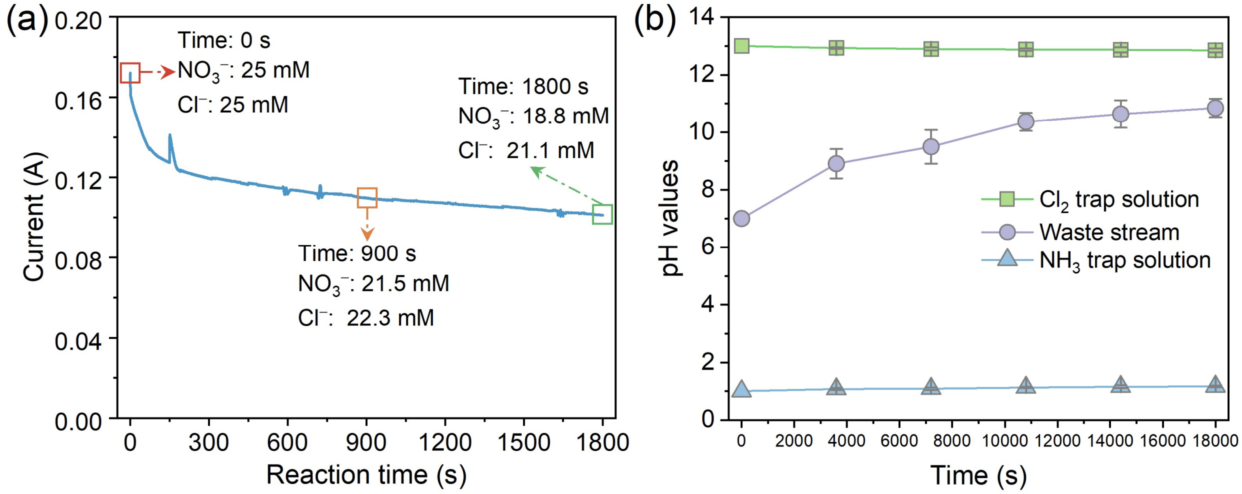

Figure S7. Stability test of the electrolyzer. (a) I-t curve and corresponding NO_3^- and Cl^- concentrations. (b) pH variations of Cl_2 trap solution, waste stream, and NH_3 trap solution. The error bars represent the standard deviations from triplicate tests.

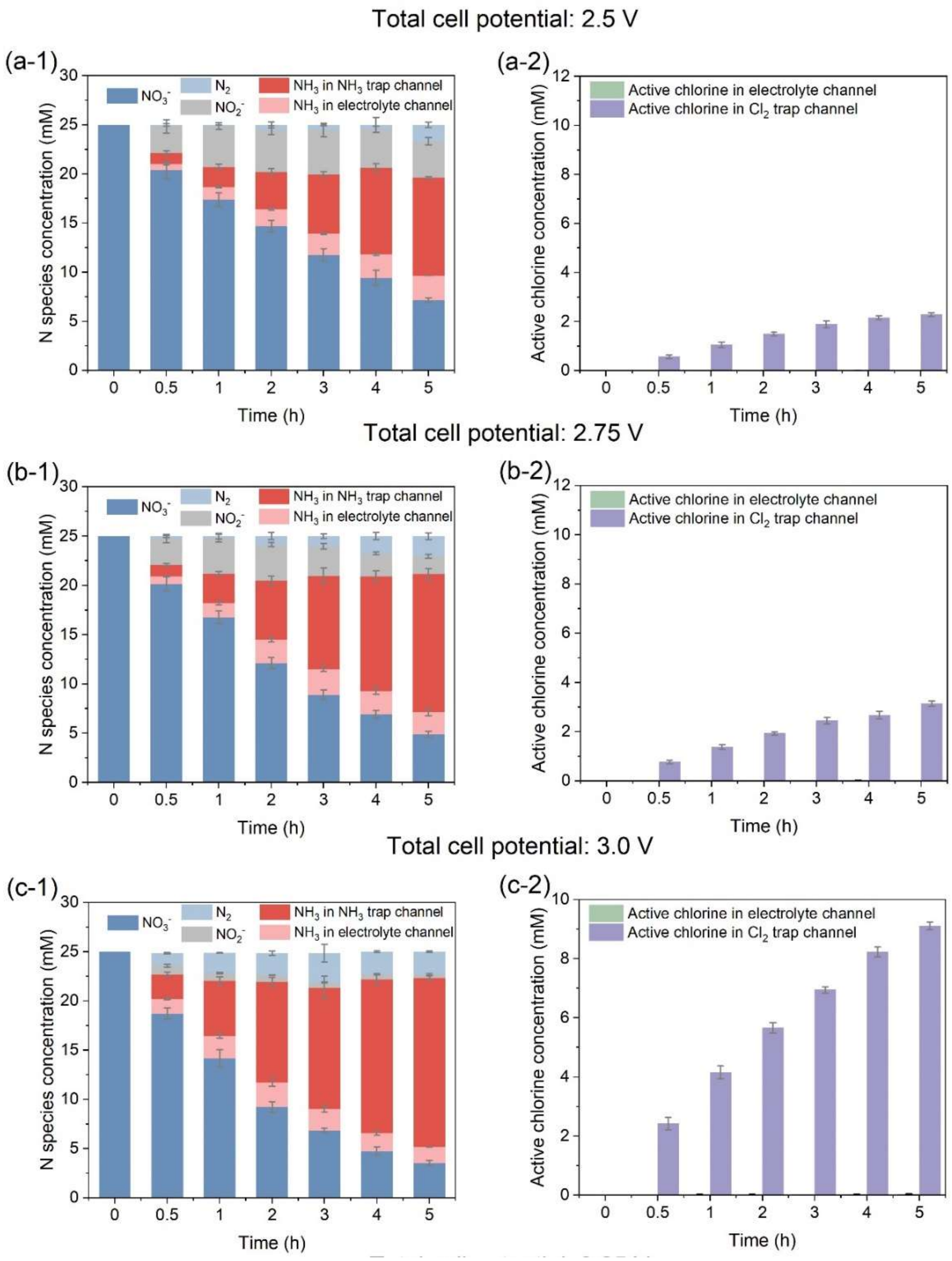
Total cell potential: 2.5 V
(a-1)
NO3-
N2
NO2-
NH3 in NH3 trap channel
NH3 in electrolyte channel
N species concentration (mM)
Time (h)
(a-2)
Active chlorine in electrolyte channel
Active chlorine in Cl2 trap channel
Active chlorine concentration (mM)
Time (h)
Total cell potential: 2.75 V
(b-1)
NO3-
N2
NO2-
NH3 in NH3 trap channel
NH3 in electrolyte channel
N species concentration (mM)
Time (h)
(b-2)
Active chlorine in electrolyte channel
Active chlorine in Cl2 trap channel
Active chlorine concentration (mM)
Time (h)
Total cell potential: 3.0 V
(c-1)
NO3-
N2
NO2-
NH3 in NH3 trap channel
NH3 in electrolyte channel
N species concentration (mM)
Time (h)
(c-2)
Active chlorine in electrolyte channel
Active chlorine in Cl2 trap channel
Active chlorine concentration (mM)
Time (h)

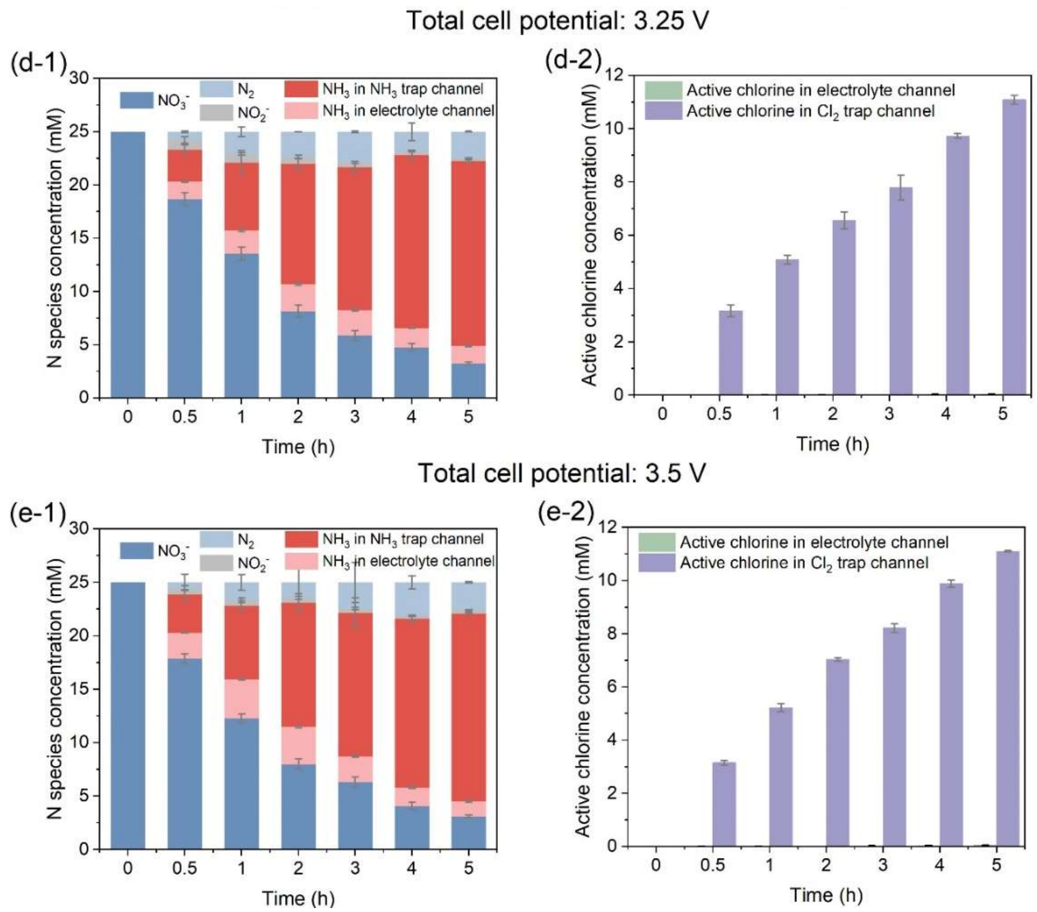

Figure S8. Nitrogen and chlorine species evolution in membrane-free electrolyzer under different cell potentials. (a) 2.5 V, (b) 2.75 V, (c) 3.0 V, (d) 3.25 V, (e) 3.5 V. Specific testing conditions: electrolyte channel: 25 mM $NaNO_3$, 25 mM NaCl, 0.1 M Na_2SO_4, pH 7.0; NH_3 trap channel: pH 1 capture solution adjusted by 1 M H_2SO_4; Cl_2 trap channel: pH 13 capture solution adjusted by 1 M NaOH. The flow rates of all electrolytes are 25 mL·min^{-1}. The error bars represent the standard deviations from triplicate tests.

Part S5. Homogeneous/heterogeneous redox reactions.

Table S1. Homogeneous/heterogeneous redox reactions related to nitrogen and chlorine species within the electrolyzer.

No.	Reactions	Rate constants or reaction potentials	Refs
	Nitrate reduction reactions		
	NO_3^- to NO_2^-		
1	$NO_3^-{}_{(ad)} + e^- \rightarrow NO_3^{2-}{}_{(ad)}$	$E^0 = -0.89$ V vs SHE	4
2	$NO_3^{2-}{}_{(ad)} + H_2O \rightarrow NO_2^{\bullet}{}_{(ad)} + 2OH^-$	$k = 5.5\times10^4\ s^{-1}$	5
3	$NO_2^{\bullet}{}_{(ad)} + e^- \rightarrow NO_2^-{}_{(ad)} + H_2O$	$E^0 = 1.04$ V vs SHE	6
	NO_2^- to NO		
4	$NO_2^-{}_{(ad)} + e^- \rightarrow NO_2^{2-}{}_{(ad)}$	$E^0 = -0.47$ V vs SHE	7
5	$NO_2^{2-}{}_{(ad)} + H_2O \rightarrow NO_{(ad)} + 2OH^-$	$k = 1.0\times10^5\ s^{-1}$	8
	NO to N_2		
**Pathway 1**			
6	$NO_{(ad)} + 2H^+ + 2e^- \rightarrow N_{(ad)} + H_2O$	E^0 is unavailable	9
7	$N_{(ad)} + N_{(ad)} \rightarrow N_2$	N.A.	9
**Pathway 2 (Vooys-Koper mechanism)**			
8	$NO_{(ad)} + NO_{(aq)} + H^+ + e^- \rightarrow HN_2O_2$	$E^0 = 0.00$ V vs SHE	10
9	$HN_2O_{2(ad)} + H^+ + e^- \rightarrow N_2O_{(ad)} + H_2O$	$E^0 = 1.59$ V vs SHE	11
10	$N_2O + e^- \rightarrow N_2O^-$	$E^0 = 1.77$ V vs SHE	12
11	$N_2O^- + 2H^+ + e^- \rightarrow N_2 + H_2O$	N.A.	13
**Pathway 3 (Duca-Feliu-Koper mechanism)**			
12	$NO_{(ad)} + 3H_2O + 4e^- \rightarrow NH_{2(ad)} + 4OH^-$	E^0 is unavailable	14
13	$NO_{(ad)} + NH_{2(ad)} \rightarrow NONH_{2(ad)}$	N.A.	14
14	$NONH_{2(ad)} \rightarrow N_2 + H_2O$	N.A.	15
	NO to NH_3		
15	$O_{(ad)} + H^+ + e^- \rightarrow HNO_{(ad)}$	$E^0 = -0.78$ V vs SHE	16
16	$HNO_{(ad)} + H^+ + e^- \rightarrow H_2NO_{(ad)}$	$E^0 = 0.52$ V vs SHE	9
17	$H_2NO_{(ad)} + H^+ + e^- \rightarrow H_2NOH_{(ad)}$	$E^0 = 0.90$ V vs SHE	17

18	$H_2NO_{(aq)} + 2H^+ + 2e^- \rightarrow NH_3 + H_2O$	$E^0 = 0.42$ V vs SHE	18
19	$NH_3 + H^+ \rightarrow NH_4^+$	pKa = 9.25	19
	Direct nitrogen species oxidation reaction		
20	$NH_3 + 3OH^- \rightarrow \frac{1}{2}N_2 + 3H_2O + 3e^-$	$E^0 = -0.77$ V vs SHE	20
	Chloride oxidation reactions		
21	$MO_x + Cl^- \rightarrow MO_x(Cl^\bullet) + e^-$ (Volmer step)	1 $mA{\cdot}cm^{-2}$: 0.2×10^{-4} s^{-1}<br>3 $mA{\cdot}cm^{-2}$: 1.3×10^{-4} s^{-1}<br>5 $mA{\cdot}cm^{-2}$: 2.3×10^{-4} s^{-1}	21
22	$MO_x(Cl^\bullet) + Cl^- \rightarrow MO_x + Cl_2 + e^-$ (Heyrovsky step)	$>1.0\times10^5$ $M^{-1}{\cdot}s^{-1}$	21
23	$MO_x(Cl^\bullet) + MO_x(Cl^\bullet) \rightarrow 2MO_x + Cl_2$ (Volmer–Tafel reaction)	1.0×10^8 $M^{-1}{\cdot}s^{-1}$	22
24	$Cl_2 + H_2O \rightarrow HOCl + H^+ + Cl^-$	0.52 $M^{-1}{\cdot}s^{-1}$	23
25	$HOCl \leftrightarrow ClO^- + H^+$	pKa = 7.5	24
	Chloride species reduction reactions		
26	$HOCl + 2e^- \rightarrow Cl^- + OH^-$	1 $mA{\cdot}cm^{-2}$: 1.8×10^{-4} s^{-1}<br>3 $mA{\cdot}cm^{-2}$: 7.5×10^{-4} s^{-1}<br>5 $mA{\cdot}cm^{-2}$: 11×10^{-4} s^{-1}	21
	Interactions between nitrogen/chloride species		
	**HOCl mediated ammonia oxidation**		
27	$NH_3 + HOCl \rightarrow NH_2Cl + H_2O$	3.1×10^6 $M^{-1}{\cdot}s^{-1}$	25
28	$NH_2Cl + HOCl \rightarrow NHCl_2 + H_2O$	1.5×10^2 $M^{-1}{\cdot}s^{-1}$	26
29	$NHCl_2 + H_2O \rightarrow NOH + 2H^+ + 2Cl^\bullet$	1.7×10^2 $M^{-1}{\cdot}s^{-1}$	27
30	$NH_2Cl + NOH \rightarrow N_2 + H^+ + Cl^- + H_2O$	8.3×10^3 $M^{-1}{\cdot}s^{-1}$	27
31	$NHCl_2 + NOH \rightarrow N_2 + 2H^+ + 2Cl^- + H_2O$	2.8×10^4 $M^{-1}{\cdot}s^{-1}$	27
32	$NHCl_2 + 2HOCl + H_2O \rightarrow NO_3^- + 5H^+ + 4Cl^-$	2.3×10^2 $M^{-1}{\cdot}s^{-1}$	28
33	$NH_3 + 4HOCl \rightarrow NO_3^- + 3H_2O + H^+ + 4Cl^-$	0.1–0.7 $M^{-1}{\cdot}s^{-1}$	21
	**HOCl/NH_2Cl mediated nitrite oxidation**		
34	$NO_2^- + HOCl \rightarrow NO_2Cl + OH^-$	$1.8\times10^5{\cdot}M^{-1}{\cdot}s^{-1}$	29
35	$H^+ + NH_2Cl + NO_2^- \rightarrow NH_3 + NO_2Cl$	unknown	
36	$NO_2Cl + NO_2^- \rightarrow N_2O_4 + Cl^-$	unknown	

37	$N_2O_4 + OH^- \rightarrow NO_3^- + NO_2^- + H^+$	fast
38	$NO_2Cl \rightarrow NO_2^+ + Cl^-$	unknown
39	$NO_2^+ + OH^- \rightarrow NO_3^- + H^+$	fast

Part S6. Detailed N-species Evolution of membrane-free electrolyzer with different NO_3^- and Cl^- concentrations.

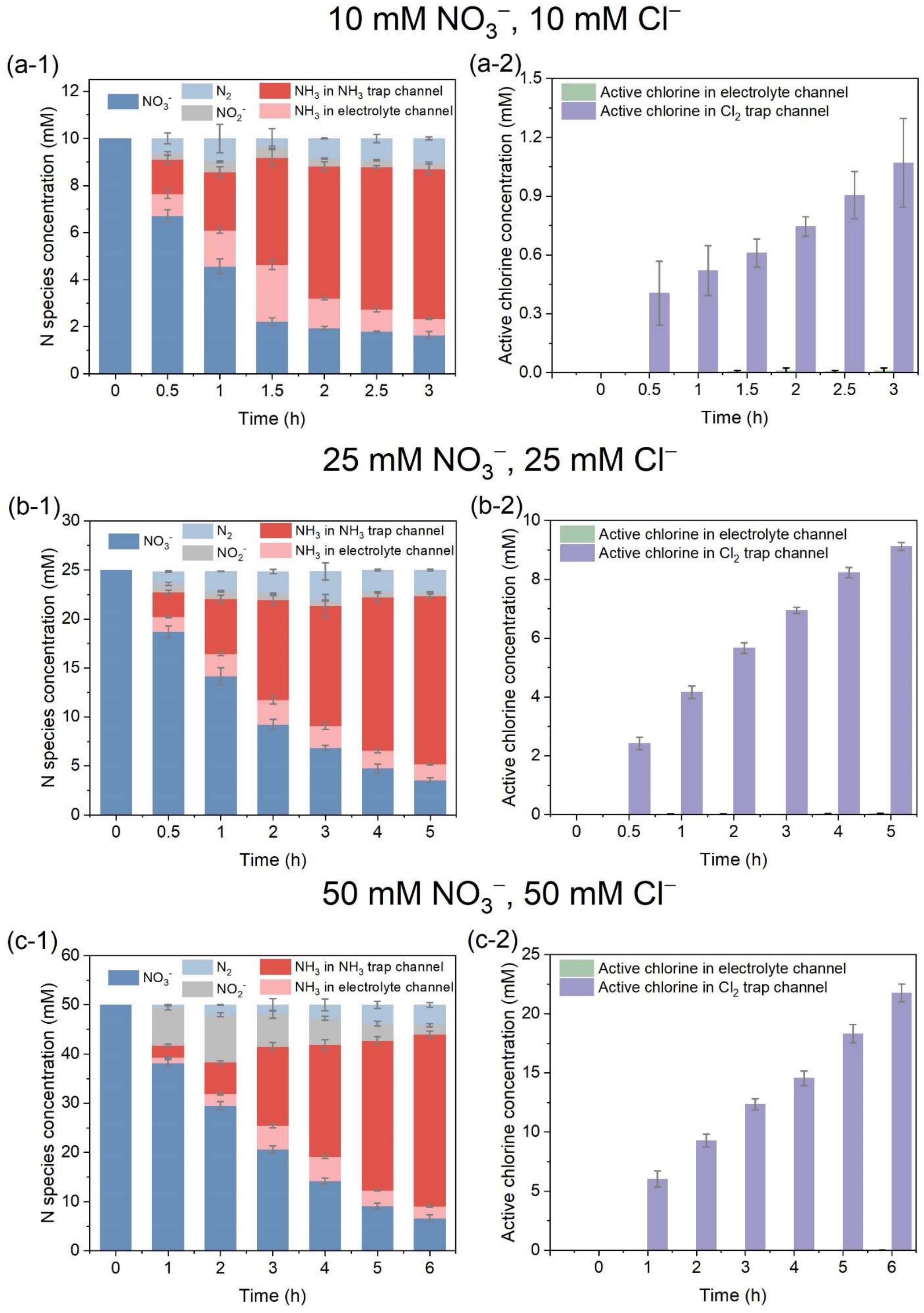

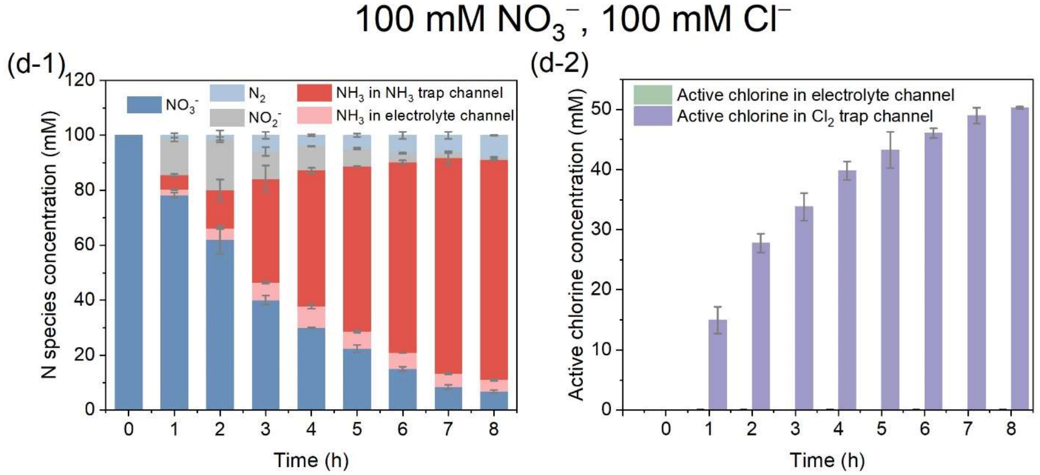

Figure S9. Nitrogen and chlorine species evolution in membrane-free electrolyzer under different nitrate and chloride concentrations. (a) 10 mM NO_3^- and 10 mM Cl^-, (b) 25 mM NO_3^- and 25 mM Cl^-, (c) 50 mM NO_3^- and 50 mM Cl^-, (d) 100 mM NO_3^- and 100 mM Cl^-. Specific testing conditions: electrolyte channel: 10-100 mM $NaNO_3$, 10-100 mM NaCl, 0.1 M Na_2SO_4, pH 7.0; NH_3 trap channel: pH 1 capture solution adjusted by 1 M H_2SO_4; Cl_2 trap channel: pH 13 capture solution adjusted by 1 M NaOH. The flow rates of all electrolytes are 25 $mL{\cdot}min^{-1}$. Total cell potentials are all set to be 3.0 V. The error bars represent the standard deviations from triplicate tests.

Part S7. Detailed N-species Evolution of membrane-free electrolyzer with different ratios of NO_3^- and Cl^-.

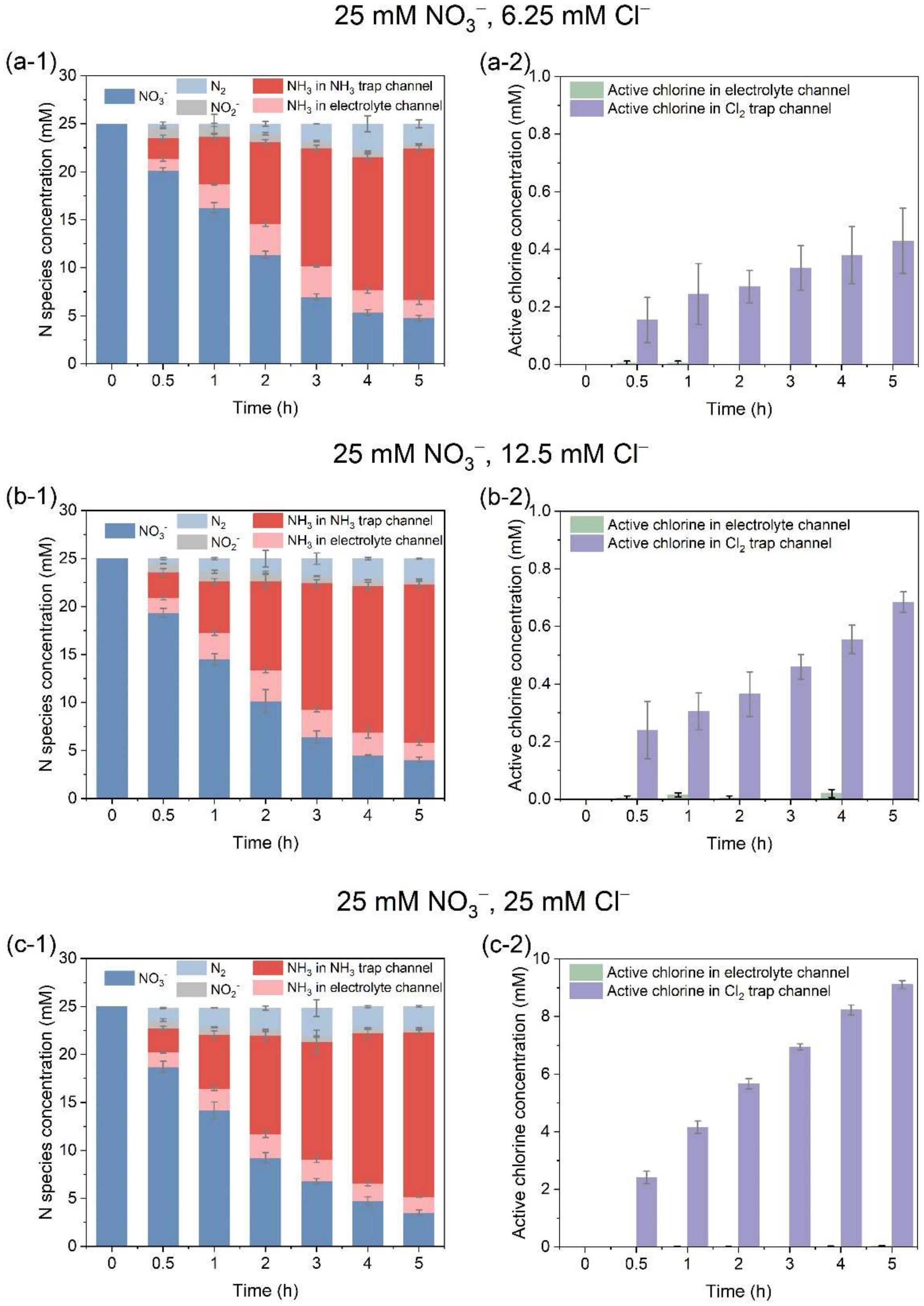

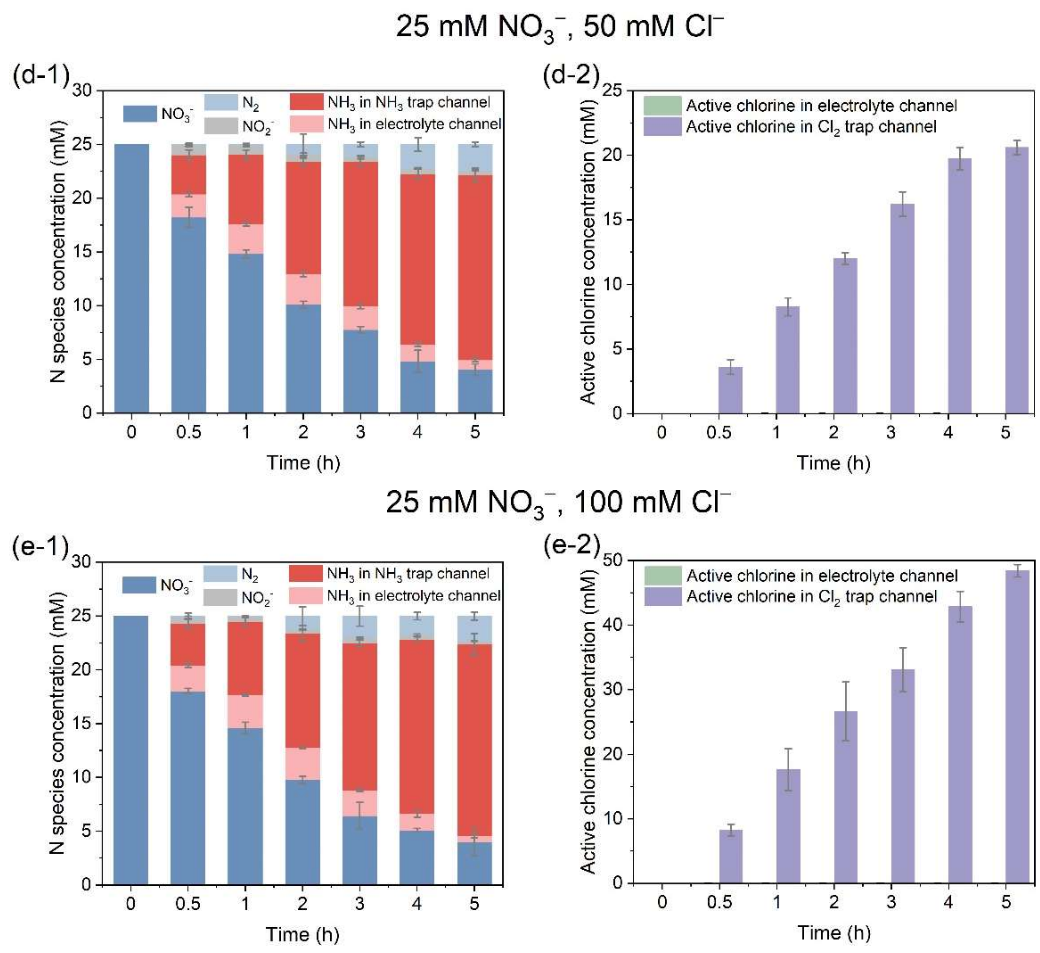

Figure S10. Nitrogen and chlorine species evolution in membrane-free electrolyzer under different ratios of NO_3^- and Cl^-. (a) 25 mM NO_3^- and 6.25 mM Cl^-, (b) 25 mM NO_3^- and 12.5 mM Cl^-, (c) 25 mM NO_3^- and 25 mM Cl^-, (d) 25 mM NO_3^- and 50 mM Cl^-, (e) 25 mM NO_3^- and 100 mM Cl^-. Specific testing conditions: electrolyte channel: 25 mM $NaNO_3$, 6.25-100 mM NaCl, 0.1 M Na_2SO_4, pH 7.0; NH_3 trap channel: pH 1 capture solution adjusted by 1 M H_2SO_4; Cl_2 trap channel: pH 13 capture solution adjusted by 1 M NaOH. The flow rates of all electrolytes are 25 mL·min^{-1}. Total cell potentials are all set to be 3.0 V. The error bars represent the standard deviations from triplicate tests.

Part S8. Summary of current densities and Faradaic Efficiencies.

Table S2. Summary of current density and NH_3/Cl_2 FE of different NO_3^- and Cl^- concentrations and ratios under the fixed 3.0 V cell potential.

Concentrations and ratios	Current densities	NH_3 FE	NO_2^- FE	N_2 FE	Cl_2 FE
NO_3^-:Cl^- concentration ratio=1					
10 mM NO_3^-, 10 mM Cl^-	4.00±0.22 mA·cm^{-2}	69.9±1.8%	0.6±0.1%	6.5±0.4%	2.7±0.6%
25 mM NO_3^-, 25 mM Cl^-	5.67±0.56 mA·cm^{-2}	79.1±1.1%	0.6±0.1%	10.1±0.4%	9.6±0.2%
50 mM NO_3^-, 50 mM Cl^-	8.44±0.67 mA·cm^{-2}	87.8±2.0%	1.1±0.2%	6.1±0.7%	25.6±0.9%
100 mM NO_3^-, 100 mM Cl^-	13.56±1.22 mA·cm^{-2}	92.6±0.5%	0.3±0.0%	7.3±0.1%	36.9±0.1%
NO_3^-:Cl^- concentration ratios from 4:1 to 1:4					
25 mM NO_3^-, 6.75 mM Cl^-	5.67±0.44 mA·cm^{-2}	74.3±0.7%	0.8±0.1%	9.1±1.8%	0.5±0.1%
25 mM NO_3^-, 12.5 mM Cl^-	5.78±0.67 mA·cm^{-2}	75.4±0.4%	0.7±0.2%	9.7±0.1%	0.7±0.0%
25 mM NO_3^-, 25 mM Cl^-	5.67±0.56 mA·cm^{-2}	79.1±1.1%	0.6±0.1%	10.1±0.4%	9.6±0.2%
25 mM NO_3^-, 50 mM Cl^-	6.78±0.33 mA·cm^{-2}	63.7±2.8%	0.6±0.1%	8.8±0.8%	18.1±0.5%
25 mM NO_3^-, 100 mM Cl^-	8.00±0.89 mA·cm^{-2}	54.7±2.5%	0.4±0.0%	7.2±1.2%	36.1±0.7%

Part S9. Techno-economic analysis

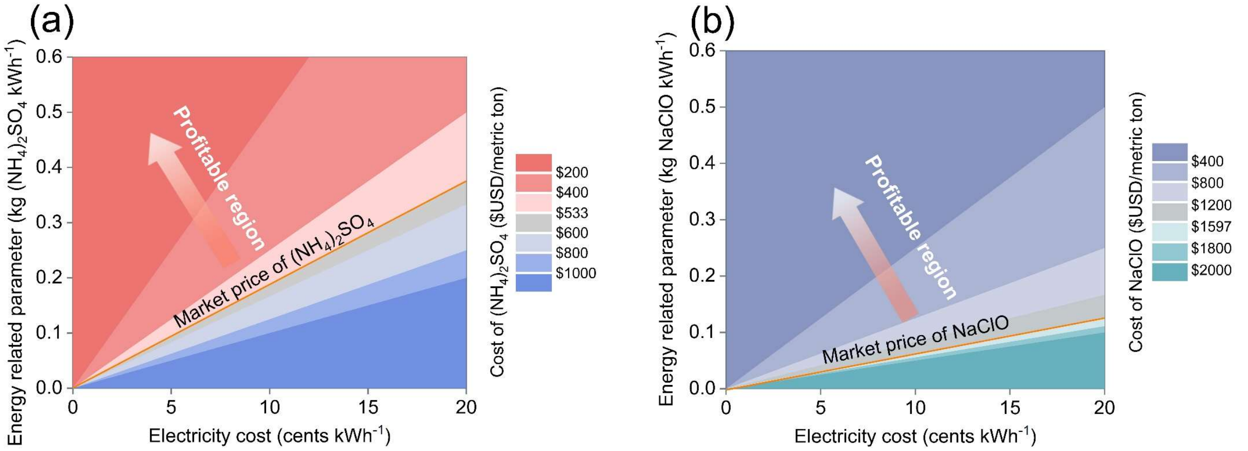

Figure S11. Techno-economic analysis. Production cost of $(NH_4)_2SO_4$ (a) and NaClO (b) as a function of energy-related parameters and unit electricity cost.

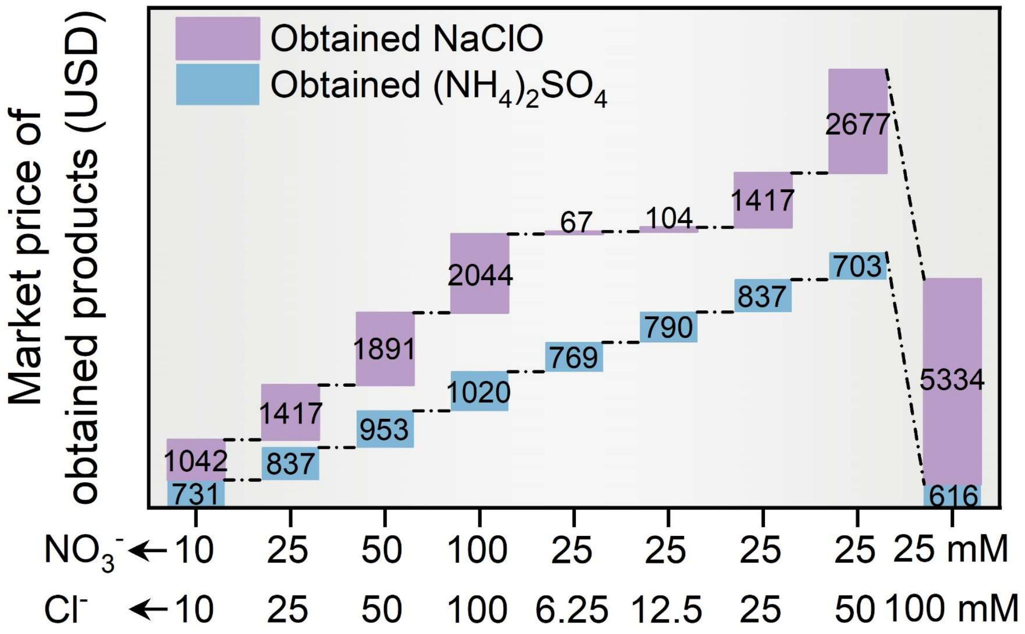

Figure S12. The market price of obtained products under 1000 USD electricity input with a set base electricity cost of 5 ¢/kWh. The calculation is based on the recovered $(NH_4)_2SO_4$ and NaClO in the NH_3/Cl_2 trap channels.

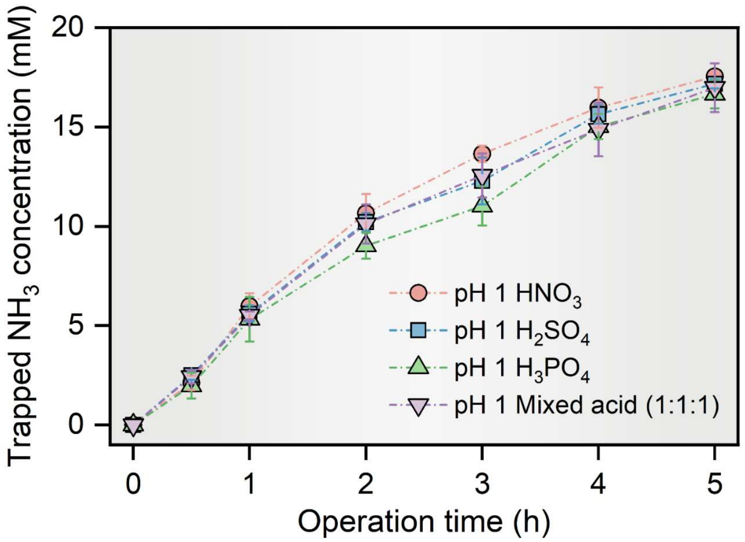

Figure S13. The comparison of various acid solution for NH_3 capture. Specific testing conditions: electrolyte channel: 25 mM $NaNO_3$, 25 mM NaCl, 0.1 M Na_2SO_4, pH 7.0; cell voltage 3.0 V. The flow rates of all electrolytes are 25 mL·min^{-1}. The error bars represent the standard deviations from triplicate tests.

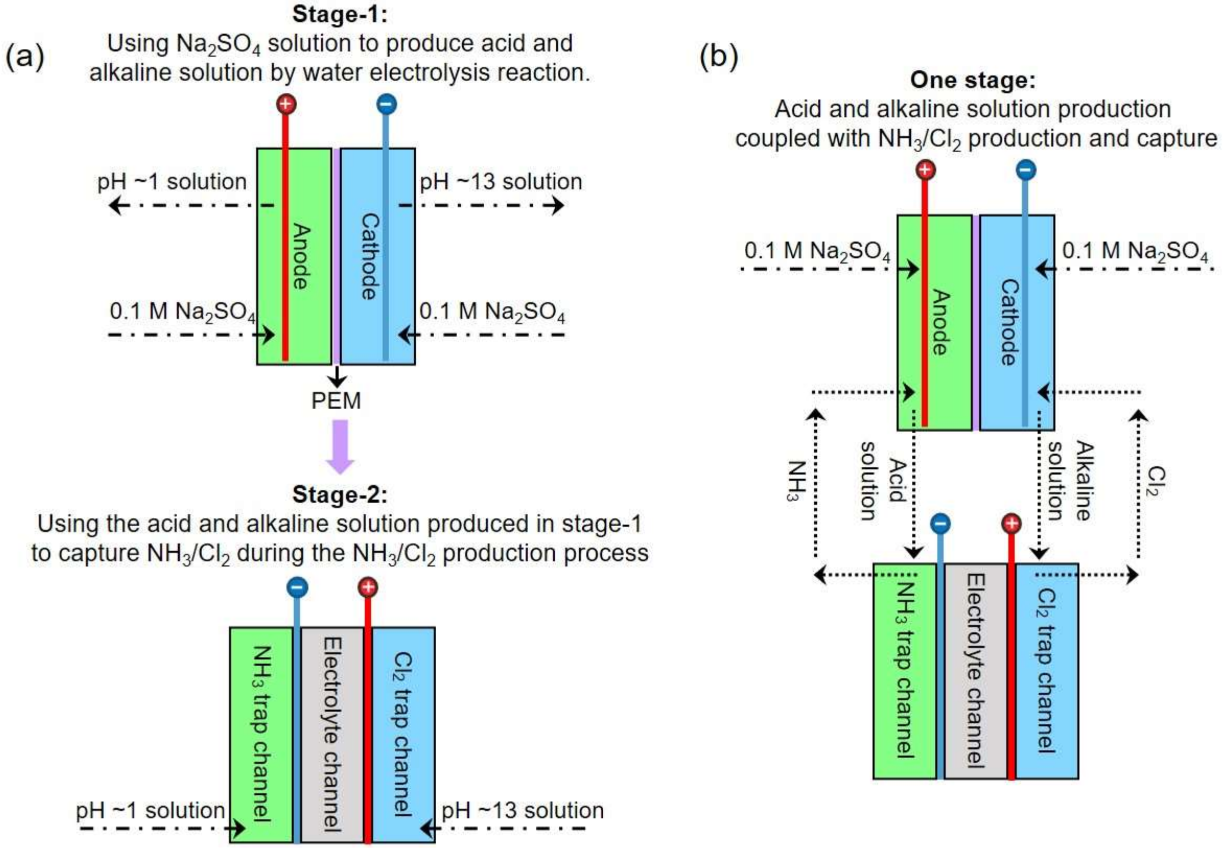

Figure S14. Illustration of different configurations. (a) two-stage operation, where the acid/base

was generated in a proton exchange membrane (PEM)-separated electrolyzer and then pumped into another electrochemical reactor for NH_3/Cl_2 separation (batch mode). (b) one-stage operation, where the acid/base was generated and continuously pumped into the electrochemical reactor for NH_3/Cl_2 separation (continuous mode).

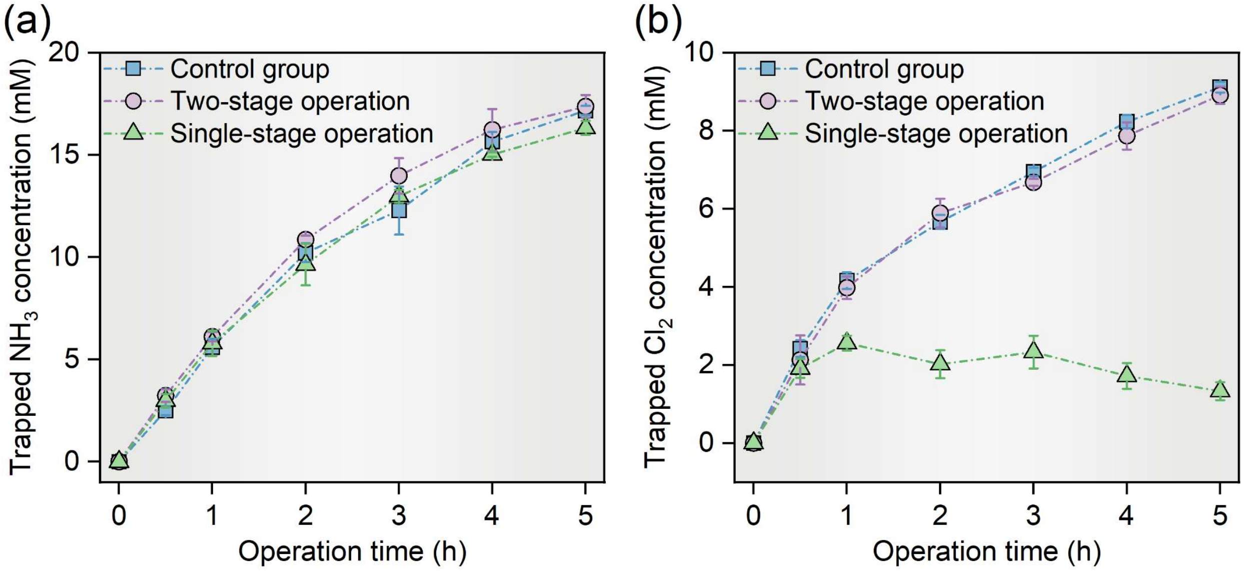

Figure S15. The comparison of two-stage operation and one-stage operation for NH_3 (a) and Cl_2 (b) capture. Specific testing conditions: electrolyte channel: 25 mM $NaNO_3$, 25 mM NaCl, 0.1 M Na_2SO_4, pH 7.0; cell voltage 3.0 V. The flow rates of all electrolytes are 25 mL·min^{-1}. The error bars represent the standard deviations from triplicate tests.

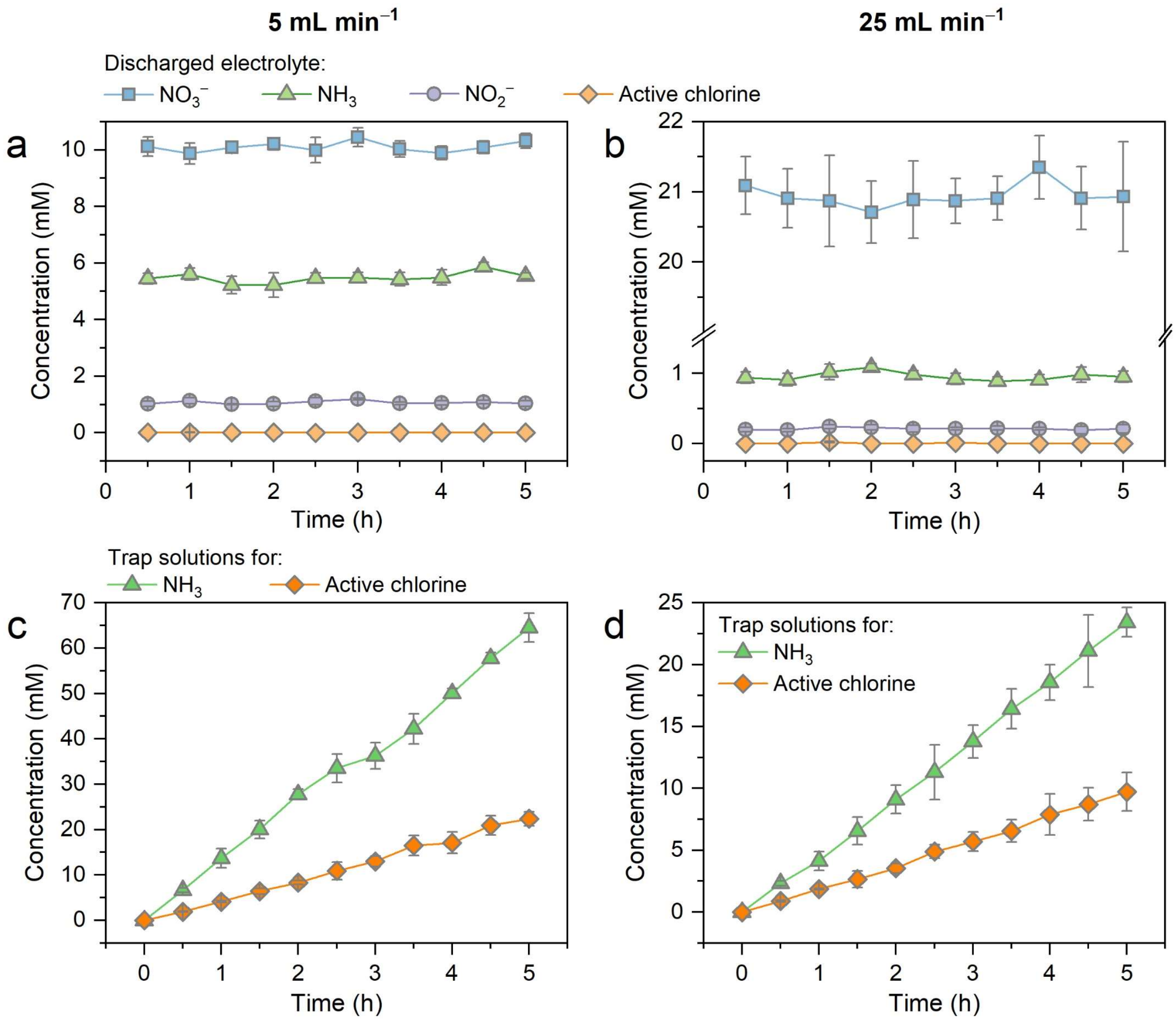

Figure S16. The single pass mode for NH_3 and Cl_2 production and separation without recirculation of the waste stream. NO_3^-, NO_2^-, NH_3, and active chlorine concentrations in discharged electrolyte for the flow rate of (a) 5 mL min^{-1} and (b) 25 mL min^{-1}. NH_3 and active chlorine concentrations in trap solution for the flow rate of (c) 5 mL min^{-1} and (d) 25 mL min^{-1}. Specific testing conditions: electrolyte channel: 25 mM $NaNO_3$, 25 mM NaCl, 0.1 M Na_2SO_4, pH 7.0; cell voltage 3.0 V. The flow rates of all electrolytes are 5 mL·min^{-1} and 25 mL·min^{-1}. The error bars represent the standard deviations from triplicate tests.

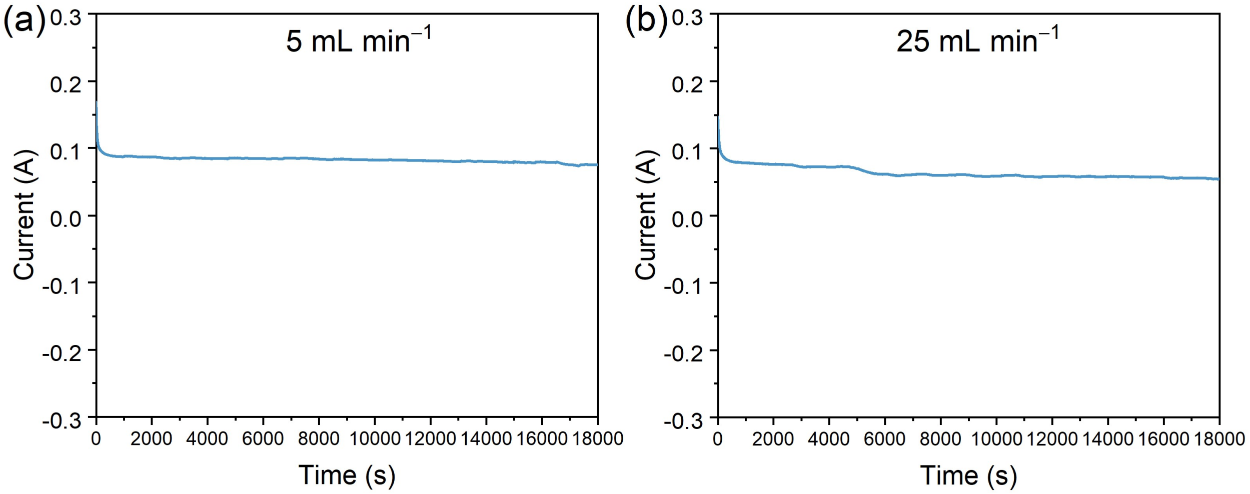

Figure S17. Typical I-t curves of single pass mode for NH_3 and Cl_2 production and separation without recirculation of the waste stream under the flow rate of (a) 5 mL min^{-1} and (b) 25 mL min^{-1} and same cell potential (3.0 V).

Part S9. Water chemistry of actual RO retentate.

Table S3. The characteristics of the obtained reverse osmosis (RO) retentate obtained from a RO plant for surface and ground water treatment.

Component	Concentration (mg L^{-1})
Conductivity	9790 uS cm^{-1}
pH	6.57 (measured at 19.5°C)
Nitrate as N	164.7 (~11.8 mM)
Aluminum	0.07
Barium	0.04
Bicarbonate as $CaCO_3$	11.3
Boron	1.47
Calcium	293
Calcium as $CaCO_3$	775
Chloride	1920 (~54.1 mM)
Chromium	0.15
Fluoride	<20
Iron	1.53
Magnesium as $CaCO_3$	650
PO_4^- as P	<20
Potassium	32.1
Silicon Dioxide	26.0
Sodium	1840
Strontium	4.24
Sulfate	2680

Part S10. Product quantification.

Electrolyte samples were collected from the trap/feed tank, diluted to the appropriate detection range, and prepared for subsequent analysis. For nitrate−N detection, 200 μl 1 M hydrochloric acid (HCl) and 20 μl 0.8 wt% sulfamic acid (H_3NSO_3) were mixed with 10.0 ml of the diluted sample. After sitting for 20 min, the absorption spectrum was measured using an ultraviolet-visible spectrophotometer and the absorption intensities at a wavelength of 220 nm and 275 nm were recorded. The final absorbance value was calculated by this equation: $A=A_{220nm}-2A_{275nm}$. The concentration-absorbance curve was calibrated using a series of standard potassium nitrate solutions and the potassium nitrate crystal was dried at 105-110 °C for 2 h in advance.

For nitrite−N detection, A mixture of 4 g p-aminobenzene sulphanilamide ($C_6H_8N_2O_2S$), 0.2 g N-(1-Naphthyl) ethylenediamine dihydrochloride ($C_{12}H_{14}N_2$), 10 mL phosphoric acid (H_3PO_4), and DI water (50 mL) was diluted to 100 mL and used as the colour reagent. Next, 200 μl colour reagent was added into 10.0 ml diluted sample and mixed well. The absorption intensity at a wavelength of 540 nm was recorded after sitting for 20 min. The concentration-absorbance curve was calibrated using a series of standard potassium nitrite solutions.

The ammonia−N concentration was spectrophotometrically detected by the indophenol blue method. In detail, 500 μl of a 1 M sodium hydroxide (NaOH) solution containing 5 wt% of salicylic acid ($C_7H_6O_3$) and 5 wt% of sodium citrate dihydrate ($C_6H_5Na_3O_7 \cdot 2H_2O$) was added into 500 μl diluted sample, followed by further addition into 250 μl of 0.05 M sodium hypochlorite (NaClO) and 50 μl of 1 wt% sodium nitroferricyanide ($C_5FeN_6Na_2O \cdot 2H_2O$) solution. The UV-Vis absorption spectrum was measured at λ = 655 nm after standing at room temperature for 2 h. The concentration-absorbance curve was calibrated using a series of standard ammonium chloride solutions.

The free chlorine (hypochlorous acid and hypochlorite ion) concentration was measured by USEPA DPD Method (HACH Method 8021). DPD free chlorine reagent powder pillow (purchased from HACH) was added to a 10 mL diluted waste sample and swirled in the sample cell for 20 seconds to mix. The UV-Vis absorption spectrum was measured at λ = 530 nm within 60 seconds of the reagent addition. The concentration-absorbance curve was calibrated using a series of

standard free chlorine solutions.

Part S11. Performance Evaluation of the NH_3 and Cl_2 production and separation.

The NO_3^-–N conversion efficiency (%) was calculated by:

$$NO_3^- - \text{N conversion efficiency} = \frac{\left[NO_3^-\right]_0 - \left[NO_3^-\right]_t}{\left[NO_3^-\right]_0} \times 100 \tag{S3}$$

where $\left[NO_3^-\right]_0$ was the initial NO_3^- concentration in electrolyte channel, $\left[NO_3^-\right]_t$ was the NO_3^- concentrations in the electrolyte channel at time t.

The Faradic efficiency (FE) (%) for NH_3 and Cl_2 were calculated by:

$$FE(\%) = \frac{n \times F \times N_i}{Q} \times 100 \tag{S4}$$

where n is the electron transfer number (8 for NH_3, 2 for NO_2^-, 5 for N_2, and 2 for Cl_2), N_i is the amount (mol) of the target products, Q is the total charge (C) passing the electrode, which was calculated based on the integration of the curve I (A) vs t (s).

The NH_3 or Cl_2 recovery efficiencies (%) were calculated by:

$$NH_3 \text{ recovery} = \frac{NH_3 \text{ concentration in } NH_3 \text{ trap channel}}{\text{Total yield } NH_3} \times 100 \tag{S5}$$

$$Cl_2 \text{ recovery} = \frac{\text{Active chlorine concentration in } Cl_2 \text{ trap channel}}{\text{Total yield active chlorine}} \times 100$$

(S6)

The product yield rates (g-$(NH_4)_2SO_4 \cdot m^{-2} \cdot d^{-1}$) were obtained by:

$$\left(NH_4\right)_2SO_4 \text{ yield rate} = \frac{m_{(NH_4)_2SO_4}}{t \times S} \tag{S7}$$

$$\text{NaClO yield rate} = \frac{m_{NaClO}}{t \times S} \tag{S8}$$

where $m_{(NH_4)_2SO_4}$ and m_{NaClO} are the mass of obtained $(NH_4)_2SO_4$ and NaClO (g) in NH_3 trap channel and Cl_2 trap channel, respectively, t is the reaction time (d), and S is the exposed size of the electrode (m^2).

The specific energy consumptions (EC) ($kWh \cdot kg^{-1}$–$(NH_4)_2SO_4$ or $kWh \cdot kg^{-1}$–NaClO) were

calculated below to evaluate the energy cost for the produced product:

$$EC = 10^{-3} \times E_{cell} \times I \times t \times m^{-1} \quad \text{(S9)}$$

where E_{cell} is the total cell potential (V), I × t is the integral area under the curve I vs t (A·h), and m is the mass of the obtained $(NH_4)_2SO_4$ or NaClO (kg).

Part S12. Reference electrode calibration.

We used saturated calomel electrode (SCE) as the reference electrode in all measurements. The calibration of SCE was carried out in a typical three-electrode system with two Pt wires serve as the working electrode and counter electrode, respectively. The electrolyte was saturated with high-purity hydrogen for at least 30 min before performing the calibration. CV was carried out at a scan rate of 1 $mV \cdot s^{-1}$ by using CH Instruments 700E Potentiostat. The average of the two potentials at which the current crossed zero was taken to be the thermodynamic potential for the hydrogen electrode reactions.[30,31]

Supplementary References

1 Yin, F. & Liu, H. The j–pH diagram of interfacial reactions involving H^+ and OH^-. *J. Energy Chem.* **50**, 339-343 (2020).

2 Gao, J. *et al.* Electrocatalytic Upcycling of Nitrate Wastewater into an Ammonia Fertilizer via an Electrified Membrane. *Environ. Sci. Technol.* **56**, 11602-11613 (2022).

3 Gao, J. *et al.* Electrochemically Selective Ammonia Extraction from Nitrate by Coupling Electron- and Phase-Transfer Reactions at a Three-Phase Interface. *Environ. Sci. Technol.* **55**, (2021).

4 Tugaoen, H. O. N., Garcia-Segura, S., Hristovski, K. & Westerhoff, P. Challenges in photocatalytic reduction of nitrate as a water treatment technology. *Sci. Total Environ.* **599-600**, 1524-1551 (2017).

5 Montesinos, V. N., Quici, N., Destaillats, H. & Litter, M. I. Nitric oxide emission during the reductive heterogeneous photocatalysis of aqueous nitrate with TiO_2. *RSC Adv.* **5**, 85319-85322 (2015).

6 da Cunha, M. C. P. M., Weber, M. & Nart, F. C. On the adsorption and reduction of NO_3^- ions at Au and Pt electrodes studied by in situ FTIR spectroscopy. *J. Electroanal. Chem.* **414**, 163-170 (1996).

7 Goldstein, S., Behar, D., Rajh, T. & Rabani, J. Nitrite Reduction to Nitrous Oxide and Ammonia by TiO_2 Electrons in a Colloid Solution via Consecutive One-Electron Transfer Reactions. *J. Phys. Chem. A* **120**, 2307-2312 (2016).

8 Su, J. F., Ruzybayev, I., Shah, I. & Huang, C. P. The electrochemical reduction of nitrate over micro-architectured metal electrodes with stainless steel scaffold. *Appl. Catal., B* **180**, 199-209 (2016).

9 Katsounaros, I. & Kyriacou, G. Influence of nitrate concentration on its electrochemical reduction on tin cathode: Identification of reaction intermediates. *Electrochim. Acta* **53**, 5477-5484 (2008).

10 de Vooys, A. C. A., Beltramo, G. L., van Riet, B., van Veen, J. A. R. & Koper, M. T. M. Mechanisms of electrochemical reduction and oxidation of nitric oxide. *Electrochim. Acta* **49**, 1307-1314 (2004).

11 de Groot, M. T. & Koper, M. T. M. The influence of nitrate concentration and acidity on the electrocatalytic reduction of nitrate on platinum. *J. Electroanal. Chem.* **562**, 81-94 (2004).

12 Zheng, J., Lu, T., Cotton, T. M. & Chumanov, G. Photoinduced Electrochemical Reduction of Nitrite at an Electrochemically Roughened Silver Surface. *J. Phys. Chem. B* **103**, 6567-6572 (1999).

13 Yang, J., Duca, M., Schouten, K. J. P. & Koper, M. T. M. Formation of volatile products during nitrate reduction on a Sn-modified Pt electrode in acid solution. *J. Electroanal. Chem.* **662**, 87-92 (2011).

14 Duca, M. *et al.* Selective Catalytic Reduction at Quasi-Perfect Pt(100) Domains: A Universal Low-Temperature Pathway from Nitrite to N_2. *J. Am. Chem. Soc.* **133**, 10928-10939 (2011).

15 Nguyen, D. A., Iwaniw, M. A. & Fogler, H. S. Kinetics and mechanism of the reaction between ammonium and nitrite ions: experimental and theoretical studies. *Chem. Eng. Sci.* **58**, 4351-4362 (2003).

16 Bartberger, M. D. *et al.* The reduction potential of nitric oxide (NO) and its importance to NO biochemistry. *Proc. Nat. Acad. Sci.* **99**, 10958-10963 (2002).

17 Dutton, A. S., Fukuto, J. M. & Houk, K. N. Theoretical Reduction Potentials for Nitrogen Oxides from CBS-QB3 Energetics and (C)PCM Solvation Calculations. *Inorg. Chem.* **44**, 4024-4028 (2005).

18 Reyter, D., Bélanger, D. & Roué, L. Elaboration by high-energy ball milling of copper/palladium composite materials – characterization and electrocatalytic activity for the reduction of nitrate in alkaline medium. *J. Electroanal. Chem.* **622**, 64-72 (2008).

19 O'Neal Tugaoen, H., Garcia-Segura, S., Hristovski, K. & Westerhoff, P. Compact light-emitting diode optical fiber immobilized TiO_2 reactor for photocatalytic water treatment. *Sci. Total Environ.* **613-614**, 1331-1338 (2018).

20 Zhang, M. *et al.* An Efficient Symmetric Electrolyzer Based On Bifunctional Perovskite Catalyst for Ammonia Electrolysis. *Adv. Sci.* **8**, 2101299 (2021).

21 Zhang, C., He, D., Ma, J. & Waite, T. D. Active chlorine mediated ammonia oxidation revisited: Reaction mechanism, kinetic modelling and implications. *Water Res.* **145**, 220-230 (2018).

22 Wu, D., Wong, D. & Di Bartolo, B. Evolution of Cl^{-}_2 in aqueous NaCl solutions. *J. Photochem.* **14**, 303-310 (1980).

23 Wang, T. X. & Margerum, D. W. Kinetics of Reversible Chlorine Hydrolysis: Temperature Dependence and General-Acid/Base-Assisted Mechanisms. *Inorg. Chem.* **33**, 1050-1055 (1994).

24 Deborde, M. & von Gunten, U. Reactions of chlorine with inorganic and organic compounds during water treatment—Kinetics and mechanisms: A critical review. *Water Res.* **42**, 13-51 (2008).

25 Qiang, Z. & Adams, C. D. Determination of Monochloramine Formation Rate Constants with Stopped-Flow Spectrophotometry. *Environ. Sci. Technol.* **38**, 1435-1444 (2004).

26 Margerum, D. W., Gray, E. T. J. & Huffman, R. P. in *Organometals and Organometalloids* Vol. 82 *ACS Symposium Series* Ch. 17, 278-291 (American Chemical Society, 1979).

27 Zhang, J. *et al.* in *Recent Advances in Disinfection By-Products* Vol. 1190 *ACS Symposium Series* Ch. 5, 79-95 (American Chemical Society, 2015).

28 Pham, H. T., Wahman, D. G. & Fairey, J. L. Updated Reaction Pathway for Dichloramine Decomposition: Formation of Reactive Nitrogen Species and N-Nitrosodimethylamine. *Environ. Sci. Technol.* **55**, 1740-1749 (2021).

29 Wahman, D. G. & Speitel, G. E., Jr. Relative Importance of Nitrite Oxidation by Hypochlorous Acid under Chloramination Conditions. *Environ. Sci. Technol.* **46**, 6056-6064 (2012).

30 Liang, Y. *et al.* Co_3O_4 nanocrystals on graphene as a synergistic catalyst for oxygen reduction reaction. *Nat. Mater.* **10**, 780-786 (2011).

31 Li, Y. *et al.* MoS_2 Nanoparticles Grown on Graphene: An Advanced Catalyst for the Hydrogen Evolution Reaction. *J. Am. Chem. Soc.* **133**, 7296-7299 (2011).
